# Federated deep reinforcement learning for privacy-preserving offloading in vehicular edge computing

**DOI:** 10.1038/s41598-026-56587-2

**Published:** 2026-06-10

**Authors:** Alaa M. Momani, Deema Mohammed Alsekait, Mahmoud Ahmad Al-Khasawneh, Siti Hajar Othman, Kholoud Alkayid, Samia Kouki, K. M. Baalamurugan

**Affiliations:** 1 School of Computing, Horizon University College, Ajman, UAE; 2 Department of Information Technology, College of Computer and Information Sciences, Princess Nourah bint Abdulrahman University, P.O. Box 84428 11671 Riyadh, Saudi Arabia; 3 Faculty of Computing, Universiti Teknologi Malaysia, 81310 Johor Bahru, Johor Malaysia; 4 Department of Computer Science, Faculty of Information Technology, Hourani Center for Applied Scientific Research, Al-Ahliyya Amman University, Amman, Jordan; 5 Computer Information Science Division, Ras Al Khaimah Campus, Higher Colleges of Technology, Ras Al Khaimah, UAE; 6 School of Engineering and IT, Manipal Academy of Higher Education, Dubai, UAE

**Keywords:** Federated learning, Deep reinforcement learning, Vehicular edge computing, Task offloading, Privacy preservation, Soft actor–critic, Engineering, Mathematics and computing

## Abstract

With the rapid development of Internet of Vehicles (IoV) applications, the demand for serving computation-intensive and delay-sensitive tasks, which are executed in a dynamic mobility environment, continues to grow, while the embedded computing power carried by vehicles remains limited, and they are facing strict requirements in terms of latency. In vehicular edge computing, to offload computation to the nearby roadside units (RSUs) and to enable centralized learning-based offloading, mobility, task, channel, and resource information at the whole system needs to be gathered at a central learner, leading to a higher communication overhead and raw data exposure. This study introduces a privacy-aware federated deep reinforcement learning (FDRL) framework for vehicular edge computing task offloading with RSU assistance. The novelty of the proposed framework does not lie in the common usage of federated learning and deep reinforcement learning (DRL), but rather in the compactness of four coupled mechanisms: generation of a hybrid action representation of federated binary offloading decision and continuous resource allocation for RSUs, a personalized federated aggregation mechanism for non-IID vehicular observations collected on the RSU, a task-criticality-aware deadline reliability model with class-dependent violation penalties, and a handover-aware multi-RSU model that incorporates signaling delay, service-context transfer delay, and processing/authentication delay. In the proposed framework, the model parameters of the local SAC policies are provided to the federated coordinator rather than the locally observed information, such as raw vehicular trajectories, which can instead be used for local training of the SAC-based policies. Controlled simulation experiments are conducted to compare the proposed method with both local execution and edge-offloading methods, two centralized DRL baselines (DQN and DDPG), and three federated DRL baselines (FedAvg-DQN, centralized-SAC, and federated-MADRL). The results indicate that under the adopted simulation settings, the proposed FDRL framework achieves competitive and/or better system cost, delay, energy, deadline-violation performance, and communication overhead compared with other schemes. This privacy usefulness really means having less raw data exposed when federated training is used, and does not mean any formal privacy guarantee against inference attacks against model updates.

## Introduction

The rapid development of intelligent transportation and the growing use of connected and autonomous vehicles have led to the introduction of the Internet of Vehicles (IoV) as one of the building blocks of next-generation smart cities^1^. The latest vehicles feature advanced sensing, communication, and computing systems, enabling a wide range of applications, including autonomous driving, real-time traffic control, and collaborative perception^2,3^. Such applications produce huge amounts of data that require real-time processing, high reliability, and low latency^4^. The energy limitations and onboard computing capabilities of vehicles, however, pose a major challenge to locally processing such data.

Mobile edge computing (MEC) has been shown to be a successful approach to addressing these problems by enabling vehicles to offload their computations to edge servers that are closer. MEC greatly reduces communication delays and enhances service responsiveness (e.g., delay-sensitive vehicular services) by bringing computing resources closer to the data source^5,6^. Consequently, the problem of efficient task offloading and scheduling has been recognized as a major concern in vehicular edge computing, aiming to optimize system performance in terms of delay, energy consumption, and resource utilization under highly dynamic network conditions^7^. Recently, deep reinforcement learning (DRL) has shown great potential for addressing complex decision-making issues in the IoV setting^8^. Using DRL to simulate adaptive offloading policies and modeling task offloading as a Markov decision process (MDP) allows DRL-based methods to adapt to time-varying channel states and heterogeneous task-to-offload policies in response to changing vehicle mobility^9^. However, most existing DRL-based offloading solutions follow a centralized training methodology, in which large volumes of raw vehicle data are collected and processed on a single server^10^. This solution poses several critical constraints, including excessive communication overhead, limited scalability, and severe privacy violations due to the disclosure of sensitive information, such as vehicle position, driving behavior, and user data.

Vehicular edge task offloading poses a challenge because decisions must be made based on time-varying channel quality, vehicle mobility, heterogeneous task urgency, limited RSU computing resources, and rigid latency requirements^11,12^. The problem is that a fixed offloading policy cannot handle these dynamically changing conditions. Centralized DRL can be trained to learn adaptive offloading policies, but this comes at the cost of aggregating system-level information, such as task attributes, vehicle mobility information, and channel-condition information, at a central learner^13,14^. Federated learning (FL) helps reduce the sharing of raw data, but on its own, it is not a solution to the inherent sequentiality of task offloading. Thus, a federated deep reinforcement learning (FDRL) architecture fits the given problem, as each RSU can learn local offloading policies in its own environment, and the federated coordinator enables RSUs to share knowledge of learnable offloading policies at the model level^15,16^. The local learner adopted in this work is SAC, as an additional change in the formulation would require the hybrid continuous action representation of the offloading propensity and edge-resource allocation.

FL has also recently been proposed as an attractive distributed learning framework that enables collaborative model training without exchanging raw data^17^. In FL, edge nodes or vehicles use local models with their own datasets and send model updates solely to a central aggregator for use in constructing the global model. However, it must be noted that FL does not necessarily bring some formal privacy guarantees. Even though the pure vehicular data will always be local, the exchanged model parameters may still leak sensitive information if they are attacked by one of the aforementioned attacks^18,19^. Thus, the privacy argument in this study is restricted to minimizing direct exposures of raw data and the communication of sensitive vehicular observations. Differential privacy, secure aggregation, and adversarial privacy-attack evaluation are not integrated into the existing framework but are listed as future extensions.

The decentralized system plays a major role in maintaining privacy and minimizing communication load^20,21^. However, task offloading in the IoV is not readily solved by the full extent of FL and DRL due to factors such as non-independent and identically distributed (non-IID) data across vehicles, high mobility, which causes dynamic client involvement, and instability of reinforcement learning in a distributed training setup^22^. Besides privacy, the available task offloading literature primarily uses average system performance indicators, such as delay and energy consumption, without adequately accounting for the essential aspects of practice in vehicle environments^23,24^. Real-world IoV systems use a heterogeneous mix of records with varying levels of urgency and importance, with safety-critical tasks (e.g., collision avoidance and object detection) requiring stringent deadline guarantees^25^. Moreover, vehicle mobility creates frequent handovers between edge servers, resulting in additional migration costs and service interruptions that should be considered in offloading decisions. When these factors are ignored, there is the risk that system behavior becomes suboptimal or even unsafe.

Despite these challenges, this study presents a privacy-aware FDRL framework for task offloading in ground RSU-assisted vehicular edge computing. However, combining FL with DRL is not novel; this has already been explored in recent work on vehicular edge computing. Rather, a key contribution is the combined design and implementation of four aspects, which are typically designed separately: SAC-based hybrid action learning for binary offloading execution and continuous RSU resource allocation; task-criticality-aware deadline reliability modeling; mobility-aware operation of multi-RSU with more accurate handover and service-context transfer costs; and personalized federated aggregation for non-IID RSU-level environments. Most current literature on offloading studies based on FDRL lacks careful consideration of all four aspects and primarily focuses at federated policy learning, mobility-aware resource optimization, or privacy-aware model sharing. Therefore, the proposed framework should be understood as an integrated formulation-level contribution rather than a claim to have invented a fundamentally new FL or reinforcement learning algorithm.

The main contributions of this study are summarized as follows:

- This study proposes a privacy-aware FDRL architecture for RSU-assisted vehicular edge computing, in which the local offloading policy of each RSU is learned based on the observed vehicular states at the vicinity of the RSU, and only the model parameters are shared with the federated coordinator, but no vehicular trajectories, tasks, mobility traces, or channel states are transmitted.
- This work proposes a hybrid SAC action representation that compensates for the discrepancy between continuous SAC learning and binary offloading execution. The actor returns a continuous sensitivity to be offloaded, and an edge-resource allocation coefficient, and the environment makes a binary decision between local/offload.
- The proposed framework adds a task-criticality-aware deadline-reliability model to the offloading objective, in which task urgency, deterministic and probabilistic deadline-violation costs, and class-dependent deadline-violation penalties are all considered.
- A mobility-aware multi-RSU edge operation model is presented, which decomposes handover delay into signaling delay, delay for transferring service context, and processing/ authentication delay, and considers service continuity during handover from one RSU to another across RSU coverage regions for the offloading policy.
- This study employs a personalized federated aggregation mechanism to balance global model sharing with local RSU adaptation under non-IID vehicular observations, improving the suitability of the learned policy for heterogeneous RSU-level operating conditions.
- An extensive set of controlled simulations is conducted against centralized and federated baselines, including DQN, DDPG, centralized SAC, FedAvg-DQN, FedAvg-DDPG, FedAvg-SAC, and Fed-MADRL, with additional analyses of convergence, system cost, delay, energy consumption, deadline violation, communication overhead, ablation behavior, personalization sensitivity, and non-IID robustness.

The rest of this paper is structured as follows. Section "Related work" reviews the area-related work on task offloading, DRL, and FL in vehicular networks. Section "System model and problem formulation "shows the system model and problem formulation. Section "Proposed federated deep reinforcement learning framework" presents the proposed FDRL. Section "Performance evaluation" presents the simulation results and performance analysis. Lastly, Section "Conclusion and future work" is the conclusion and future research directions.

## Related work

Task offloading from VECs has been the most studied solution to address the limited computational power of onboard vehicle devices. Cloud offloading was proposed in^26^, in which a computation task is sent to remote cloud servers. However, the research of^27^ found that these solutions exhibit excessive latency and cannot be used as delay-critical components in IoV systems. Recent research^28^ on MEC has focused on deploying edge servers closer to vehicles to reduce latency and improve service delivery. Equally, the author in^29^ proposed optimization-based task offloading schemes for simultaneous tasks, considering delay, energy consumption, and resource assignment. Besides, a number of studies^30,31^ modeled the offloading problem as a mixed-integer optimization problem, and these methods tend to be computationally intensive. Moreover, as mentioned in^32^, such approaches are not particularly adequate for the highly dynamic vehicles. To address these drawbacks, heuristic and metaheuristic approaches have been investigated, with^33^ using greedy algorithms and^34^ using genetic algorithms. However, these models are not flexible and do not capture the long-term dynamics of the systems.

DRL is a recent, powerful tool in dynamic decision-making in vehicular networks. Task offloading was considered in^35^ as a MDP, allowing agents to acquire optimal policies over time. MC-2PF further demonstrates the importance of personalized FL for predicting multi-edge loads^36^. In^37^, the task offloading study used deep Q-network (DQN), but DQN is not capable of operating in a high-dimensional, continuous action space. To address this drawback, more recent studies^38,39^ have adopted actor-critic algorithms such as deep deterministic policy gradient (DDPG) for continuous control tasks. Similarly, the author in^40^ demonstrated that DRL can effectively enhance system performance in dynamic network settings. However, most current DRA solutions rely on centralized training models that consolidate information from multiple vehicles at a central server. In addition, this centralized paradigm suffers from scalability issues and high communication overheads, as claimed in^41^. Also, it raises profound privacy issues, including the disclosure of sensitive vehicle information, as noted in^42^. Furthermore, there is considerable literature that emphasizes methods to optimize both average delay and energy usage, but does not consider the task-criticality and reliability criteria of safety-critical systems^43^.

FL is one approach proposed to address privacy concerns in distributed learning. To facilitate joint model training without having direct access to raw vehicular data^44^ utilized FL to support joint model training without necessarily having access to raw vehicular data. Conversely, the current study aims at offload computation tasks and allocate resources to RSU-assisted vehicular edge computing, considering SAC-based hybrid action learning, task-criticality-constrained deadline penalties, refined modeling of handover delays, and personalized federated aggregation. In^45^, the study established that FL can significantly reduce communication overhead while maintaining data privacy. Recent studies^46,47^ have used FL in edge computing and vehicular networks for distributed resource management. On the same note, the author in^48^ also investigated FL-based task offloading strategies in IoVs. However, reinforcement learning is not fully utilized to support dynamic decision-making, as most current studies rely on supervised learning. Besides, implementing FL alongside DRL raises additional issues, including non-independent and identically distributed (non-IID) data, slow convergence, and inefficient communication^49,50^. In addition, these difficulties are more critical in the context of IoVs, which are characterized by high mobility, dynamic topology, and device heterogeneity^51^.

There are some recent studies that are closely related to the current study. A study by^52^ on dynamic vehicular offloading used federated continuous reinforcement learning, whilst^53^ proposed privacy-aware task offloading based on federated multi-agent reinforcement learning^54^ proposed jointly federated DRR with game-theoretic decision making, and^55^ analyzed multi-agent collaboration in offloading vehicular tasks as part of FDRL. Those studies support the relevance of federated reinforcement learning for cooperatively adapting to the vehicle offloading task; however, the probability of deadline violation, task urgency, the cost of handover, and non-IID RSU adaptation have not been formulated as core aspects of the offloading goal. The study that is most similar to the current one is the privacy-aware hierarchical reinforcement learning-based task offloading in low-altitude vehicular fog computing proposed by^56^. Their model is UAV-assisted low-altitude VFC through the hierarchical federated reinforcement learning, AFedPPO, and contextual clustering/personalized FL^57^. Conversely, the current study comprises ground RSU-based vehicular edge computing and develops offloading strategies based on SAC-based hybrid control, task criticality, risk of deadline violation, energy-delay trade-off, and handover-aware service continuity.

Recent studies have also made advances in adaptive offloading and resource management in heterogeneous edge environments. proposed resilient collaborative caching to support robust federated deep learning. and for service migration in multi-edge IoV systems, with mobility prioritized. Moreover, studies on personalized federated DRL, actor-critic resource allocation, and workload prediction also contribute to the adaptive decision-making of distributed edge systems . The proposed framework outperforms these studies by combining SAC-based hybrid offloading/resource management, task-criticality-sensitive deadline penalties, refined handover-delay modeling, and personalized federated aggregation of non-IID RSU-level data. Although much has been done, several key problems remain. First, current DRL-based solutions are often centralized in training, which can limit scalability and raise privacy concerns . Second, existing FL-based procedures have yet to be fully incorporated into reinforcement learning for sequential decision-making problems. Third, the majority of research fails to account for task heterogeneity, deadline pressure, and reliability requirements in safety-critical applications . Lastly, the influence of vehicle mobility, such as handover cost and task migration across RSUs, is usually simplified or omitted.

Several recent studies are closely related to the present work as they already investigate federated reinforcement learning, mobility-aware vehicular offloading, privacy-aware edge intelligence and personalized federated optimization^58^. For instance, this approach has been applied to dynamic vehicular offloading via federated continuous reinforcement learning^59,60^ or has been studied for privacy-aware federated multi-agent reinforcement learning, game-theoretic federated resource allocation, or hierarchical federated reinforcement learning in vehicular fog and edge computing. These works validate the relevance of federated reinforcement learning in the case of distributed vehicular offloading. However, they typically focus on a single or set of two aspects of the problem, distributed policy learning^61,62^ or personalized aggregation, privacy-aware model sharing, mobility-aware resource allocation. In contrast, the problem investigated by this work has not been done by previous works; the problem of simultaneous and joint hybrid binary-continuous offloading control along with task-criticality-aware deadline reliability, handover-aware service continuity guarantee and personalized adaptation under non-IID RSU observations.

The distinction is crucial as vehicular edge offloading isn’t a distributed learning issue. A practical RSU-assisted vehicular edge system should determine whether to offload or not a specific task, how much edge resource to allocate when choosing to offload, how to impose a penalty for violating the task’s deadline for safety-critical systems, how handover and service-context transfer impact the execution delay, and how edge resource usage should be adaptable when the traffic density, channel quality, workload intensity and criticality of the tasks vary between different RSUs. Previous studies using the FDRL approach have not consistently derived such dimensions into a consolidated offloading objective.

Further clarity into the positioning with existing studies is provided by summarizing Table 1 in which the positioning of the proposed framework is summarized with respect to representative research streams related to federated and DRL-based vehicular edge offloading. The comparison shows that prior work has focused on related components, including federated offloading, DRL-based resource allocation, mobility-aware optimization, and personalized FL. However, these parts are oftentimes discussed individually or with various characteristics of the systems. In the current work, hybrid SAC-based binary/continuous offloading, criticality-aware deadline reliability, refined handover-aware multi-RSU operation, and personalization aggregation are jointly integrated into a single RSU-level FDRL formulation.

**Table 1:** Positioning of the proposed framework relative to existing vehicular edge offloading studies.

Research stream	Main emphasis	Remaining limitation	Distinction of this work
Conventional VEC offloading optimization	Delay, energy, and resource-allocation optimization using mathematical, heuristic, or metaheuristic methods	Limited adaptability under highly dynamic mobility, channel variation, and stochastic task arrivals	Uses SAC-based adaptive policy learning under dynamic vehicular edge conditions
Centralized DRL-based VEC offloading	Sequential offloading decisions using DQN, DDPG, SAC, or related DRL methods	Requires centralized observations and may increase communication overhead and raw-data exposure	Uses RSU-level federated learning so that raw vehicular observations remain local
Federated learning-based vehicular offloading	Distributed model training with reduced raw-data transmission	Often focuses on model sharing or supervised/federated optimization without fully modeling sequential offloading control	Integrates federated learning with SAC-based sequential offloading and resource-allocation decisions
Federated DRL-based offloading	Collaborative DRL training across distributed vehicular or edge agents	Frequently omits explicit task criticality, probabilistic deadline reliability, refined handover cost, or RSU-level personalization as a unified objective	Jointly models hybrid SAC action learning, deadline reliability, handover-aware operation, and personalized aggregation
Mobility-aware vehicular edge intelligence	Mobility, caching, service migration, or handover-aware edge operation	Mobility is often handled separately from federated DRL-based offloading and safety-critical deadline modeling	Incorporates handover delay, service-context transfer, and task migration cost directly into the offloading objective
Personalized federated learning approaches	Adaptation to non-IID client or edge-node data	Personalization is usually not combined with hybrid SAC offloading, task-criticality-aware reliability, and multi-RSU handover modeling	Uses personalized aggregation to balance global policy sharing with local RSU adaptation under non-IID observations
Proposed FDRL framework	Privacy-aware RSU-level collaborative learning, hybrid offloading/resource allocation, deadline reliability, handover-aware operation, and non-IID personalization	Not a formal privacy-guaranteed framework because differential privacy, secure aggregation, and adversarial privacy testing are not included	Provides an integrated formulation-level contribution for safety-sensitive ground RSU-assisted VEC task offloading

## System model and problem formulation

**Fig. 1:**
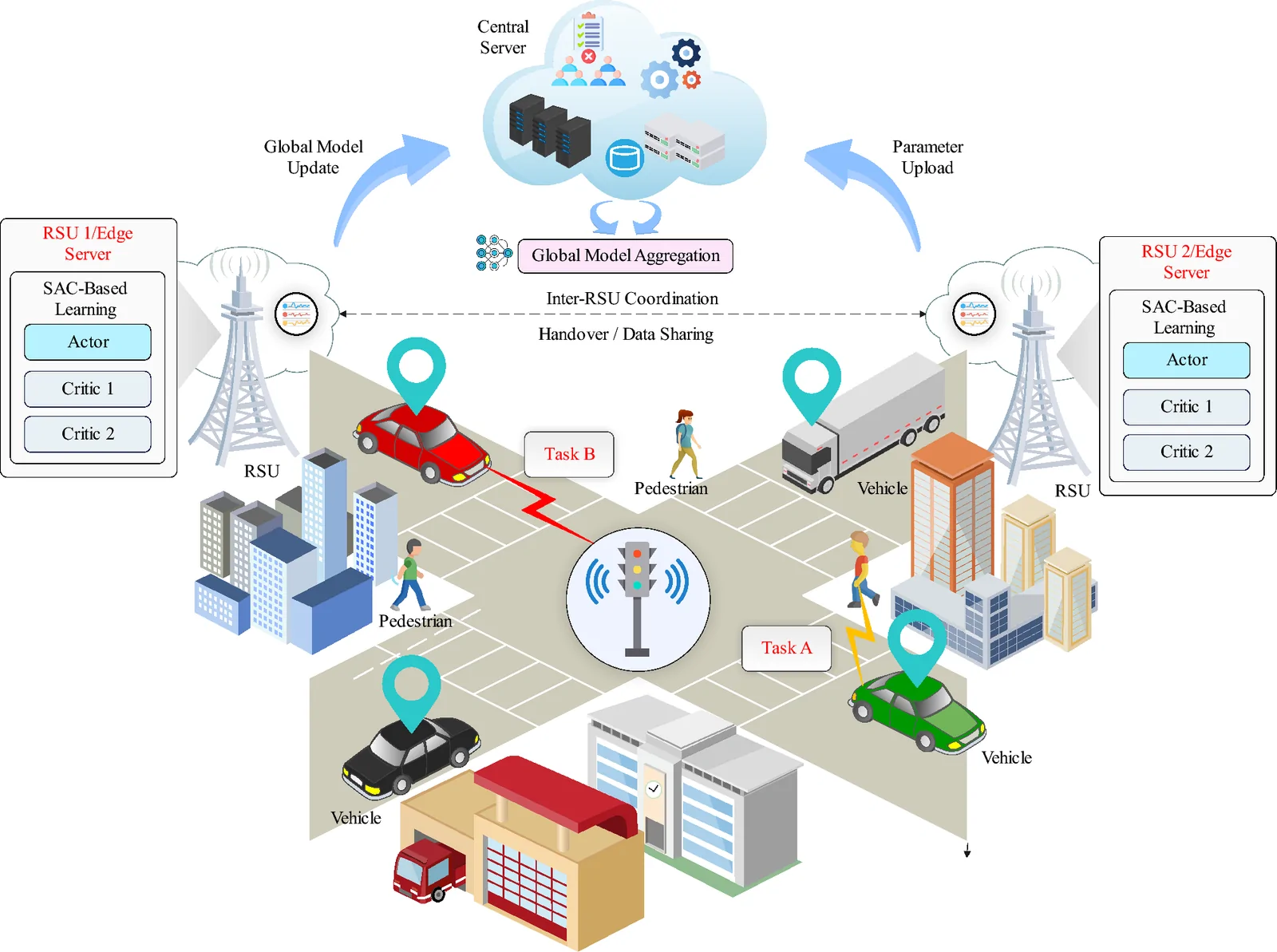
Proposed FDRL-based SAC-enabled task offloading framework for vehicular edge computing.

This section presents the system model and formulates the privacy-aware task offloading problem in vehicular edge computing. We first describe the vehicular edge architecture, followed by the task model, hybrid SAC action representation, communication model, computation model, mobility-aware handover model, and deadline-reliability formulation. The optimization problem is then defined and reformulated as a MDP to support reinforcement-learning-based solution design. Fig. 1 illustrates the proposed FDRL-based SAC-enabled task offloading framework.

We assume that an edge computing system based on a vehicular network configuration consists of a group of vehicles, a group of road side units (RSUs) with edge servers, and an FL coordinator. The set of vehicles is represented by $\mathcal {V}=\{1,2,\ldots ,N\}$, and the set of RSUs is represented by $\mathcal {R}=\{1,2,\ldots ,K\}$. Both vehicles dynamically issue computation-intensive tasks, which can be executed locally on the vehicle or offloaded to a serving RSU for execution at the edge.

Each time slot is linked to one serving RSU based on its location, communication range and channel condition. As vehicles are mobile, the associated RSU can change over time, leading to dynamic network topology, handover costs, and service migration overhead. The RSUs are viewed as fixed infrastructure nodes, whereas the vehicle-RSU association changes with vehicle mobility. Each RSU has a local reinforcement learning agent that monitors the local vehicular space and trains task offloading decisions using locally available data. To maintain data privacy, raw automobile data, including vehicle position, task, mobility trace, and channel observations, are not sent to a main server. Each RSU, instead, only exchanges model parameters with the FL coordinator. The coordinator integrates the received parameters to develop a global policy and repackages the new model for the RSUs. The architecture will facilitate joint policy learning and minimize privacy leakage and communication overhead.

Each vehicle $i \in \mathcal {V}$ generates computation tasks over discrete time slots. The task generated by vehicle *i* at time slot *t* is represented in Eq. (1) as

1 $$\begin{aligned} \mathcal {T}_i^t = \left( D_i^t, c_i^t, \tau _i^t, \chi _i^t\right) , \end{aligned}$$

where $D_i^t$ denotes the input data size in bits, $c_i^t$ denotes the required number of CPU cycles per bit, $\tau _i^t$ denotes the task deadline, and $\chi _i^t$ denotes the task criticality class. The criticality class is defined as

2 $$\begin{aligned} \chi _i^t \in \{\text {critical},\text {delay-sensitive},\text {best-effort}\}. \end{aligned}$$

Critical tasks are a safety-oriented implementation in vehicles, including collision warning, cooperative perception, and emergency braking assistance. Delay-sensitive tasks are those that cannot be delayed without impacting the entire system’s functionality: real-time navigation, traffic state updates, display of rear data, etc. Delay-sensitive tasks are those that cannot be delayed without affecting the overall functionality of the system: real-time navigation, traffic state updates, display of rear data, etc. That classification Eq. (2) distinguishes the time and resources that are demanded by a computation $c_i^t$ and the degree of urgency emphasized by a task $\chi _i^t$ without the confusion introduced by the fact that both the demand in computing resources and the demand in semantic urgency are designated by the same symbol. The deadline in which the task should be done $\tau _i^t$ and the criticality class $\chi _i^t$ both determine the reliability requirement of the task. Critical tasks are assigned stricter punishments for violations of the deadline than delay-sensitive and best-effort tasks. This enables the offloading policy to prioritize safety-sensitive vehicular applications in the event of limited communication or edge computing resources.

The task offloading problem is a mixed decision process since the end implementation option is binary, and the resource allocation is non-binary. Thus, the policy proposed based on the SAC distinguishes the continuous action of the actor network from the executable offloading decision applied in the vehicular edge environment. For each vehicle *i* at time slot *t*, the SAC actor first produces a continuous offloading propensity score $\rho _i^t \in [0,1]$, where a larger value indicates a stronger preference for edge offloading. The corresponding executable binary offloading decision is defined in Eq. (3) as

3 $$\begin{aligned} a_i^t ={\left\{ \begin{array}{ll}1, & \rho _i^t \ge 0.5,\\ 0, & \rho _i^t < 0.5,\end{array}\right. }\end{aligned}$$

where $a_i^t=0$ indicates local execution on the vehicle and $a_i^t=1$ indicates task offloading to the associated RSU. During training, $a_i^t$ is sampled from $\textrm{Bernoulli}(\rho _i^t)$ to preserve stochastic exploration, whereas during evaluation the deterministic threshold rule is applied. In addition to the offloading propensity, the actor outputs a continuous edge-resource allocation coefficient, $\eta _i^t \in [0,1]$, for each offloaded task. Accordingly, the local SAC action for vehicle *i* is represented in Eq. (4) as

4 $$\begin{aligned} \textbf{u}_i^t = [\rho _i^t,\eta _i^t],\end{aligned}$$

where $\rho _i^t$ determines the final binary offloading decision and $\eta _i^t$ controls the relative proportion of RSU computing resources assigned to the task. This hybrid action representation resolves the mismatch between binary offloading execution and continuous SAC learning, because SAC learns over the continuous action vector $\textbf{u}_i^t$ while the environment executes the binary decision $a_i^t$.

For clarity, Table 2 summarizes the main notation used in the system model and optimization formulation. The symbol *\varepsilon* denotes the maximum tolerable probability of deadline violation, while $\epsilon _0$ is used only as a small positive numerical constant to avoid division by zero in resource allocation. The coefficients *λ* and $\mu _{\chi _i^t}$ control the relative importance of energy consumption and deadline violation in the optimization objective. The coefficient $\mu _{\chi _i^t}$ is criticality-dependent, so that stricter penalties can be assigned to safety-critical tasks.

**Table 2:** Main notation used in the system model.

Symbol	Definition	Symbol	Definition
$\mathcal {V}$	Set of vehicles	$\mathcal {R}$	Set of RSUs
*N*	Number of vehicles	*K*	Number of RSUs
$\mathcal {T}_i^t$	Task generated by vehicle *i* at time slot *t*	$D_i^t$	Input data size of task $\mathcal {T}_i^t$
$c_i^t$	Required CPU cycles per bit	$\tau _i^t$	Task deadline
$\chi _i^t$	Task criticality class	$\rho _i^t$	Continuous offloading propensity generated by SAC
$\eta _i^t$	Continuous edge-resource allocation coefficient	$a_i^t$	Executable binary offloading decision
$\textbf{u}_i^t$	Hybrid SAC action for vehicle *i*	$R_{i,k}^t$	V2I transmission rate between vehicle *i* and RSU *k*
$T_i^{\textrm{tx},t}$	Uplink transmission delay	$T_i^{\textrm{rx},t}$	Result feedback delay from RSU to vehicle
$T_i^{\textrm{loc},t}$	Local computation delay	$T_i^{\textrm{edge},t}$	Edge execution delay
$k_i^{t-1}$, $k_i^t$	Previous and current serving RSUs of vehicle *i*	$T_i^{\textrm{handover},t}$	Total handover/service migration delay
$T_i^{\textrm{sig},t}$	Signaling delay during RSU reassociation	$T_i^{\textrm{ctx},t}$	Service-context transfer delay between previous and current RSUs
$T_i^{\textrm{proc},t}$	Processing, authentication, and control-plane delay during handover	$S_{\textrm{sig}}$	Signaling-message size during handover
$S_{\textrm{ctx}}$	Service-context size transferred between RSUs	$R_{k_i^{t-1},k_i^t}^{\textrm{bh},t}$	Backhaul transmission rate between previous and current RSUs
$B_{\textrm{bh}}$	Backhaul bandwidth	$P_{\textrm{bh}}$	Backhaul transmission power
$h_{k_i^{t-1},k_i^t}^{\textrm{bh},t}$	Backhaul channel gain between previous and current RSUs	$\sigma _{\textrm{bh}}^2$	Backhaul noise power
*δ*	Average handover delay coefficient in the simplified constant-delay case	$E_i^{\textrm{loc},t}$	Local computation energy consumption
$E_i^{\textrm{edge},t}$	Edge execution energy consumption	$I_i^t$	Deadline-violation indicator
*\varepsilon*	Maximum tolerable deadline-violation probability	*λ*	Energy-consumption weight
$\mu _{\chi _i^t}$	Criticality-dependent deadline-violation penalty	*β*	Personalization coefficient used in federated aggregation

Vehicles communicate with RSUs through wireless vehicle-to-infrastructure (V2I) links. Let $k_i^t \in \mathcal {R}$ denote the RSU associated with vehicle *i* at time slot *t*. The achievable uplink transmission rate between vehicle *i* and its serving RSU is defined in Eq. (5) as

5 $$\begin{aligned} R_{i,k_i^t}^t = B \log _2 \left( 1+\frac{P_i h_{i,k_i^t}^t}{\sigma ^2} \right) , \end{aligned}$$

where *B* denotes the channel bandwidth, $P_i$ is the transmission power of vehicle *i*, $h_{i,k_i^t}^t$ represents the channel gain between vehicle *i* and RSU $k_i^t$, and $\sigma ^2$ is the noise power. Based on the achievable transmission rate, the uplink transmission delay for offloading task $\mathcal {T}_i^t$ is given in Eq. (6) as

6 $$\begin{aligned} T_i^{\textrm{tx},t} = \frac{D_i^t}{R_{i,k_i^t}^t}. \end{aligned}$$

The transmission delay depends on the task input size and the instantaneous V2I channel condition. The result feedback delay from the RSU to the vehicle is denoted by $T_i^{\textrm{rx},t}$. Since the output result is typically smaller than the input task data, $T_i^{\textrm{rx},t}$ is modeled as a small non-zero delay term to avoid underestimating the end-to-end edge execution latency.

A generated task can either be executed locally on the vehicle or offloaded to the associated RSU for edge execution. If task $\mathcal {T}_i^t$ is executed locally, the local computation delay and local computation energy consumption are defined in Eqs. (7) and (8), respectively:

7 $$\begin{aligned} T_i^{\textrm{loc},t} = \frac{D_i^t c_i^t}{f_i^{\textrm{loc}}}, \end{aligned}$$

8 $$\begin{aligned} E_i^{\textrm{loc},t} = \kappa \left( f_i^{\textrm{loc}}\right) ^2 D_i^t c_i^t, \end{aligned}$$

where $f_i^{\textrm{loc}}$ denotes the CPU frequency of vehicle *i*, and *κ* is the effective switched-capacitance coefficient of the processor. When task $\mathcal {T}_i^t$ is offloaded to RSU $k_i^t$, the RSU assigns part of its available computing capacity to the task. Let $\mathcal {V}_{k}^{t}$ denote the set of vehicles offloading tasks to RSU *k* at time slot *t*. For an offloaded task, the allocated edge CPU frequency is determined by the continuous allocation coefficient $\eta _i^t$, as shown in Eq. (9):

9 $$\begin{aligned} f_{i,k_i^t}^{\textrm{edge},t} = \frac{\eta _i^t}{\sum _{j \in \mathcal {V}_{k_i^t}^{t}} \eta _j^t+\epsilon _0} f_{k_i^t}^{\max }, \end{aligned}$$

where $f_{k_i^t}^{\max }$ is the maximum computing capacity of RSU $k_i^t$, and $\epsilon _0$ is a small positive constant used to avoid division by zero. The edge execution delay includes uplink transmission delay, edge computation delay, and result feedback delay. The corresponding edge execution delay and energy consumption are defined in Eqs. (10) and (11), respectively:

10 $$\begin{aligned} T_i^{\textrm{edge},t} = T_i^{\textrm{tx},t} + \frac{D_i^t c_i^t}{f_{i,k_i^t}^{\textrm{edge},t}} + T_i^{\textrm{rx},t}, \end{aligned}$$

11 $$\begin{aligned} E_i^{\textrm{edge},t} = P_i T_i^{\textrm{tx},t} + \kappa \left( f_{i,k_i^t}^{\textrm{edge},t}\right) ^2 D_i^t c_i^t. \end{aligned}$$

In the equation Eq. (11), the first term is the uplink transmission energy, and the second one is the edge computation energy. This formulation ensures that the binary offloading decision, together with the continuous resource allocation in the RSU, influences the delay and energy cost, respectively.

Due to vehicle mobility, the serving RSU of a vehicle may change between consecutive time slots. Let $k_i^{t-1}$ and $k_i^t$ denote the previous and current serving RSUs of vehicle *i*, respectively. A handover occurs when $k_i^t \ne k_i^{t-1}$. In practical vehicular edge computing, handover delay is not only a fixed switching penalty; it may also depend on signaling overhead, service context transfer between RSUs, and processing or authentication delays. Therefore, the handover delay is modeled in Eq. (12) as

12 $$\begin{aligned} T_i^{\textrm{handover},t} = \mathbb {I}\left( k_i^t \ne k_i^{t-1}\right) \left( T_i^{\textrm{sig},t} + T_i^{\textrm{ctx},t} + T_i^{\textrm{proc},t} \right) , \end{aligned}$$

where $\mathbb {I}(\cdot )$ is the indicator function, $T_i^{\textrm{sig},t}$ denotes the signaling delay for RSU reassociation, $T_i^{\textrm{ctx},t}$ denotes the service-context transfer delay between the previous and current RSUs, and $T_i^{\textrm{proc},t}$ denotes the processing, authentication, and control-plane delay during handover. The signaling delay, context-transfer delay, and RSU-to-RSU backhaul rate are defined in Eqs. (13–15), respectively:

13 $$\begin{aligned} T_i^{\textrm{sig},t} = \frac{S_{\textrm{sig}}}{R_{i,k_i^t}^{t}}, \end{aligned}$$

14 $$\begin{aligned} T_i^{\textrm{ctx},t} = \frac{S_{\textrm{ctx}}}{R_{k_i^{t-1},k_i^t}^{\textrm{bh},t}}, \end{aligned}$$

15 $$\begin{aligned} R_{k_i^{t-1},k_i^t}^{\textrm{bh},t} = B_{\textrm{bh}} \log _2 \left( 1+ \frac{ P_{\textrm{bh}}h_{k_i^{t-1},k_i^t}^{\textrm{bh},t} }{\sigma _{\textrm{bh}}^2} \right) . \end{aligned}$$

Here, $S_{\textrm{sig}}$ is the signaling-message size, $R_{i,k_i^t}^{t}$ is the V2I transmission rate between vehicle *i* and its current serving RSU, $S_{\textrm{ctx}}$ is the service-context size, $R_{k_i^{t-1},k_i^t}^{\textrm{bh},t}$ is the backhaul transmission rate between the previous and current RSUs, $B_{\textrm{bh}}$ is the backhaul bandwidth, $P_{\textrm{bh}}$ is the RSU backhaul transmission power, $h_{k_i^{t-1},k_i^t}^{\textrm{bh},t}$ is the backhaul channel gain, and $\sigma _{\textrm{bh}}^2$ is the backhaul noise power. This refined handover formulation allows the handover cost to vary with the V2I channel condition, the RSU-to-RSU backhaul quality, and the context-transfer overhead. When detailed signaling and backhaul information are unavailable, the handover model can be reduced to the simplified average-delay form in Eq. (16):

16 $$\begin{aligned} T_i^{\textrm{handover},t} = \delta \cdot \mathbb {I}\left( k_i^t \ne k_i^{t-1}\right) , \end{aligned}$$

where *δ* is the mean handover delay coefficient. Therefore, the handover-delay model is considered a simplified special case among the main mobility models.

In this study, the term multi-RSU collaboration is used to refer to federated coordination and handover-related service-context transfer, as opposed to constant peer-to-peer exchange of raw data about vehicles with other RSUs. RSUs collaborate at the learning level by providing locally trained model parameters to the federated coordinator. Moreover, as a vehicle switches its serving RSU, the prior and current RSUs exchange service-context information using the backhaul link and this cost is incurred $T_i^{\textrm{ctx},t}$. Hence, RSU collaboration is enabled through federated aggregation and context transfer in handover-aware situations. The total end-to-end execution delay and total energy consumption of task $\mathcal {T}_i^t$ are defined in Eqs. (17) and (18), respectively:

17 $$\begin{aligned} T_i^t = (1-a_i^t)T_i^{\textrm{loc},t} + a_i^t \left( T_i^{\textrm{edge},t} + T_i^{\textrm{handover},t} \right) , \end{aligned}$$

18 $$\begin{aligned} E_i^t = (1-a_i^t)E_i^{\textrm{loc},t} + a_i^t E_i^{\textrm{edge},t}. \end{aligned}$$

Therefore, local execution consists of local computation delay and energy used, whereas edge execution consists of uplink transmission, edge computation, feedback of result, potential delay due to handover, and related edge energy consumption. In order to model the deadline reliability, the deadline-violation indicator and probabilistic deadline constraint are presented in Eqs. (19) and (20), respectively:

19 $$\begin{aligned} I_i^t = {\left\{ \begin{array}{ll} 1, & T_i^t > \tau _i^t,\\ 0, & T_i^t \le \tau _i^t. \end{array}\right. } \end{aligned}$$

20 $$\begin{aligned} \Pr \left( T_i^t > \tau _i^t\right) \le \varepsilon , \end{aligned}$$

where $\tau _i^t$ is the task deadline and *\varepsilon* denotes the maximum tolerable probability of missing the deadline. A smaller value of *\varepsilon* represents stricter reliability requirements and is especially relevant for safety-critical vehicular applications.

According to the system model above, the task offloading objective is to minimize the expected total system cost, including execution delay, energy consumption, and deadline-violation penalty. The optimization problem is formulated in Eq. (21) as

21 $$\begin{aligned} \min \ \mathbb {E} \left[ \sum _{i \in \mathcal {V}} \left( T_i^t + \lambda E_i^t + \mu _{\chi _i^t} I_i^t \right) \right] , \end{aligned}$$

where $T_i^t$ is the total task execution delay, $E_i^t$ is the total energy consumption, and $I_i^t$ is the deadline-violation indicator. The coefficient *λ* controls the relative importance of energy consumption, while $\mu _{\chi _i^t}$ denotes the criticality-dependent deadline-violation penalty associated with the task class $\chi _i^t$. To reflect different reliability requirements among vehicular tasks, the deadline penalty is assigned according to task criticality as defined in Eq. (22) as:

22 $$\begin{aligned} \mu _{\textrm{critical}}> \mu _{\mathrm {delay-sensitive}} > \mu _{\mathrm {best-effort}}. \end{aligned}$$

This environment prescribes that missed deadlines for safety-critical tasks incur a more severe penalty than for non-urgent tasks, enabling the policy to address the priority of critical applications in limited cases of communication or computing resources. The optimization problem is subject to the constraints in Eqs. (23–27):

23 $$\begin{aligned} a_i^t&\in \{0,1\}, & \forall i \in \mathcal {V}, \end{aligned}$$

24 $$\begin{aligned} \rho _i^t&\in [0,1], & \forall i \in \mathcal {V}, \end{aligned}$$

25 $$\begin{aligned} \eta _i^t&\in [0,1], & \forall i \in \mathcal {V}, \end{aligned}$$

26 $$\begin{aligned} \Pr \left( T_i^t > \tau _i^t\right)&\le \varepsilon , & \forall i \in \mathcal {V}, \end{aligned}$$

27 $$\begin{aligned} \sum _{i \in \mathcal {V}_{k}^{t}} f_{i,k}^{\textrm{edge},t}&\le f_k^{\max }, & \forall k \in \mathcal {R}. \end{aligned}$$

The first constraint characterizes the decision to perform physical binary offloading, and the second and third constraints characterize the continuous SAC outputs of that decision. The fourth constraint is the probabilistic deadline-reliability requirement, and the final constraint is to ensure that the sum of the allocated edge CPU frequency in each RSU does not exceed its computing capacity. Thus, the structure of the problem at the final point of execution is discrete-continuous in nature: the final decision of the execution is discrete, but the offloading propensity, as the continuous component, and the allocation of resources are continuous quantities.

The task offloading problem is dynamic, stochastic, and non-convex in nature since over time the mobility of the vehicles, the quality of wireless channels, the arrival of tasks, and the availability of the resources of RSUs vary. So, we formulate the problem of a MDP. At each time slot *t*, the system state is represented in Eq. (28) as

28 $$\begin{aligned} s_t = \left\{ \textbf{m}^t, \textbf{h}^t, \textbf{q}^t, \textbf{g}^t \right\} , \end{aligned}$$

where $\textbf{m}^t$ represents vehicle mobility and RSU association information, $\textbf{h}^t$ represents V2I channel states, $\textbf{q}^t$ represents task features including data size, CPU cycles, deadline, and criticality class, and $\textbf{g}^t$ represents available RSU computing resources. Based on the observed state, the SAC agent selects the continuous action vector, and the corresponding executable binary offloading vector is obtained from the offloading propensity, as defined in Eqs. (29) and (30):

29 $$\begin{aligned} \textbf{u}^t = \left\{ [\rho _1^t,\eta _1^t], [\rho _2^t,\eta _2^t], \ldots , [\rho _N^t,\eta _N^t] \right\} , \end{aligned}$$

30 $$\begin{aligned} \textbf{a}^t = \{a_1^t,a_2^t,\ldots ,a_N^t\}. \end{aligned}$$

Here, $\rho _i^t$ is the offloading propensity, $\eta _i^t$ is the edge-resource allocation coefficient for vehicle *i*, and each $a_i^t$ is obtained from $\rho _i^t$ using Bernoulli sampling during training and deterministic thresholding during evaluation. Thus, the SAC policy is trained using the continuous action representation $\textbf{u}^t$, while the vehicular edge environment executes the corresponding binary offloading decisions $\textbf{a}^t$. The immediate reward is defined in Eq. (31) as the negative weighted system cost:

31 $$\begin{aligned} r_t = - \sum _{i \in \mathcal {V}} \left( T_i^t + \lambda E_i^t + \mu _{\chi _i^t} I_i^t \right) . \end{aligned}$$

This reward motivates the agent to minimize latency levels, energy usage and breaches of deadlines on important tasks simultaneously with the agent being excited by stronger fines imposed upon deadline violations on critical tasks through $\mu _{\chi _i^t}$. Using a constrained-MDP penalty relaxation addresses the probabilistic deadline constraint in Eq. (20). In particular, violations of deadlines are punished with the help of the criticality-dependent term $\mu _{\chi _i^t}I_i^t$, which helps the SAC agent to avoid actions that repeatedly violate task deadlines. During the evaluation process, the probability of deadline violations, determined empirically, is computed and compared with *\varepsilon*. Thus, the empirical enforcement of the chance constraint is achieved by reward penalty and verification of the reliability of the post-training type of performance, instead of through closed-form analytical constraint satisfaction at all available time steps.

Under the suggested FL system, three RSUs conduct each local policy training using local observed transitions of their state. A global model is built by pooling the local model parameters rather than raw vehicular data to facilitate collaborative learning and minimize direct exposure to raw data. The global model parameters are computed in Eq. (32) as

32 $$\begin{aligned} \theta ^{(g)} = \sum _{k=1}^{K} \frac{n_k}{n} \theta _k, \end{aligned}$$

where $\theta _k$ denotes the local model parameters at RSU *k*, $n_k$ is the number of local training samples at RSU *k*, and $n=\sum _{k=1}^{K}n_k$ is the total number of training samples across all RSUs. Personalized federated aggregation using coefficient *β* is described in Section "Proposed federated deep reinforcement learning framework".

The above formulation summarizes the key issues of task offloading in vehicular edge computing, such as latency, energy efficiency, mobility-induced handover, deadline reliability, and privacy-aware distributed learning. The fact that the resulting problem is characterized by stochastic system dynamics, binary execution decisions, continuous resource allocation, and coupled RSU resource constraints makes it difficult to address it efficiently using simple generalized optimization techniques. This is why the next section of this paper presents the proposed FDRL scheme for learning scalable, privacy-aware offloading policies.

## Proposed federated deep reinforcement learning framework

This section outlines the suggested FDRL architecture pertaining to privacy-aware task offloading to vehicular edge computing. In forming the framework, FL is incorporated with the Soft Actor-Critic (SAC) algorithm to enable distributed policy learning across various RSUs without passing raw vehicular data to a central learner. A local SAC agent trained by each RSU using locally perceived vehicle states, task data, channel state, and resource provisioning. The RSUs now and then update model parameters in their files and send them to the federated coordinator, which aggregates the received parameters and redistributes the updated overall model. It is a design that allows collaborative learning with minimal exposure to raw data and reduced communication overhead. The framework is structured around three key components: local policy learning, which trains a SAC-based offloading policy at each RSU; federated model aggregation; and local personalization, which learns an IID RSU-level non-IID offloading policy. In this regard, the proposed framework is not a generic alternative for existing FDRL methods; instead, it focuses on specializing FDRL for RSU-assisted vehicular edge computing, where hybrid SAC action learning, task-criticality-aware reliability, handover-aware service continuity, and non-IID RSU personalization are co-optimized in a single offloading framework.

To address the discrete-continuous nature of the offloading problem, SAC is implemented using the continuous action representation described above. The actor network produces, for each vehicle, an offloading propensity $\rho _i^t \in [0,1]$ and a resource allocation coefficient $\eta _i^t \in [0,1]$. Bernoulli sampling during training is converted into the executable binary decision $a_i^t$, whereas $\eta _i^t$ directly controls the edge-resource allocation of offloaded tasks. Thus, SAC is not used directly as a binary-action direct relaxation solver; it instead learns a continuous relaxation between the offloading decision and resource allocation. This architecture retains the ability to execute binary tasks and enjoys the entropy-regularized exploration and stability of continuous policy learning. The SAC objective function is given in Eq. (33) as

33 $$\begin{aligned} J(\pi ) = \sum _{t} \mathbb {E}_{(s_t,\textbf{u}_t)\sim \pi } \left[ r(s_t,\textbf{u}_t) + \alpha \mathcal {H}\big (\pi (\cdot \mid s_t)\big ) \right] , \end{aligned}$$

where $\textbf{u}_t$ denotes the continuous SAC action, *α* is the entropy temperature parameter controlling the trade-off between exploration and exploitation, and $\mathcal {H}(\pi )$ denotes the policy entropy. The SAC model is a jointly optimized combination of actor and critic networks that learn stable policies in continuous decision spaces. This critic estimates the soft Q-function by minimizing the Bellman residual and maximizing the expected return, with entropy regularization. The corresponding critic loss, target value, actor objective, and entropy-temperature objective are defined in Eqs. (34)–(37):

34 $$\begin{aligned} L_Q(\theta ) = \mathbb {E} \left[ \left( Q_\theta (s_t,\textbf{u}_t) - y_t \right) ^2 \right] , \end{aligned}$$

35 $$\begin{aligned} y_t = r_t + \gamma \mathbb {E}_{\textbf{u}_{t+1}\sim \pi } \left[ Q_{\theta '}(s_{t+1},\textbf{u}_{t+1}) - \alpha \log \pi (\textbf{u}_{t+1} \mid s_{t+1}) \right] , \end{aligned}$$

36 $$\begin{aligned} J_\pi (\phi ) = \mathbb {E}_{s_t} \left[ \mathbb {E}_{\textbf{u}_t \sim \pi _\phi } \left[ \alpha \log \pi _\phi (\textbf{u}_t \mid s_t) - Q_\theta (s_t,\textbf{u}_t) \right] \right] , \end{aligned}$$

37 $$\begin{aligned} J(\alpha ) = \mathbb {E}_{\textbf{u}_t \sim \pi } \left[ -\alpha \log \pi (\textbf{u}_t \mid s_t) - \alpha \mathcal {H}_{\textrm{target}} \right] . \end{aligned}$$

In these formulations, *θ* and *ϕ* denote the critic and actor network parameters, respectively, while *θ '* denotes the target critic parameters. The discount factor $\gamma \in (0,1)$ controls the importance of future rewards. The entropy-regularized actor objective promotes exploration, which is particularly useful in dynamic vehicular scenarios where channel quality, mobility, task arrivals, and RSU resources vary over time. The adaptive entropy-temperature-parameter update also further balances exploration and exploitation during training, allowing local SAC agents to learn robust offloading and resource-allocation policies under retractable vehicular edge conditions.

Each RSU $k \in \mathcal {R}$ operates as an independent learning agent and maintains an actor network $\pi _{\phi _k}$ together with two critic networks, $Q_{\theta _k}^{1}$ and $Q_{\theta _k}^{2}$, to improve training stability. At each time step, the RSU observes the current system state $s_t$, and the local actor network outputs a continuous action vector $\textbf{u}^t \sim \pi _{\phi _k}(s_t)$. This vector contains the offloading propensity $\rho _i^t$ and the resource allocation coefficient $\eta _i^t$ for each vehicle associated with RSU *k*. The offloading propensity is then converted into the executable binary decision $a_i^t$: during training, $a_i^t$ is sampled from $\textrm{Bernoulli}(\rho _i^t)$ to preserve exploration, whereas during evaluation it is obtained using the deterministic threshold rule $a_i^t=\mathbb {I}(\rho _i^t \ge 0.5)$.

The resulting binary offloading decision and continuous resource allocation coefficient are executed in the environment. The reward $r_t$ is then computed according to the resulting delay, energy consumption, and deadline-violation penalty. The transition tuple $(s_t,\textbf{u}^t,r_t,s_{t+1})$ is stored in the replay buffer, and the local SAC networks are updated using mini-batches sampled from this buffer. It is important to note that the replay buffer stores the continuous SAC action $\textbf{u}^t$, not only the final binary offloading vector. The binary variable $a_i^t$ is used for environment execution and delay/energy calculation, whereas the actor and critic are trained using the continuous action representation. This prevents a mismatch between the binary offloading formulation and the SAC learning procedure. The local training procedure is summarized in Algorithm 1.

**Algorithm 1:**
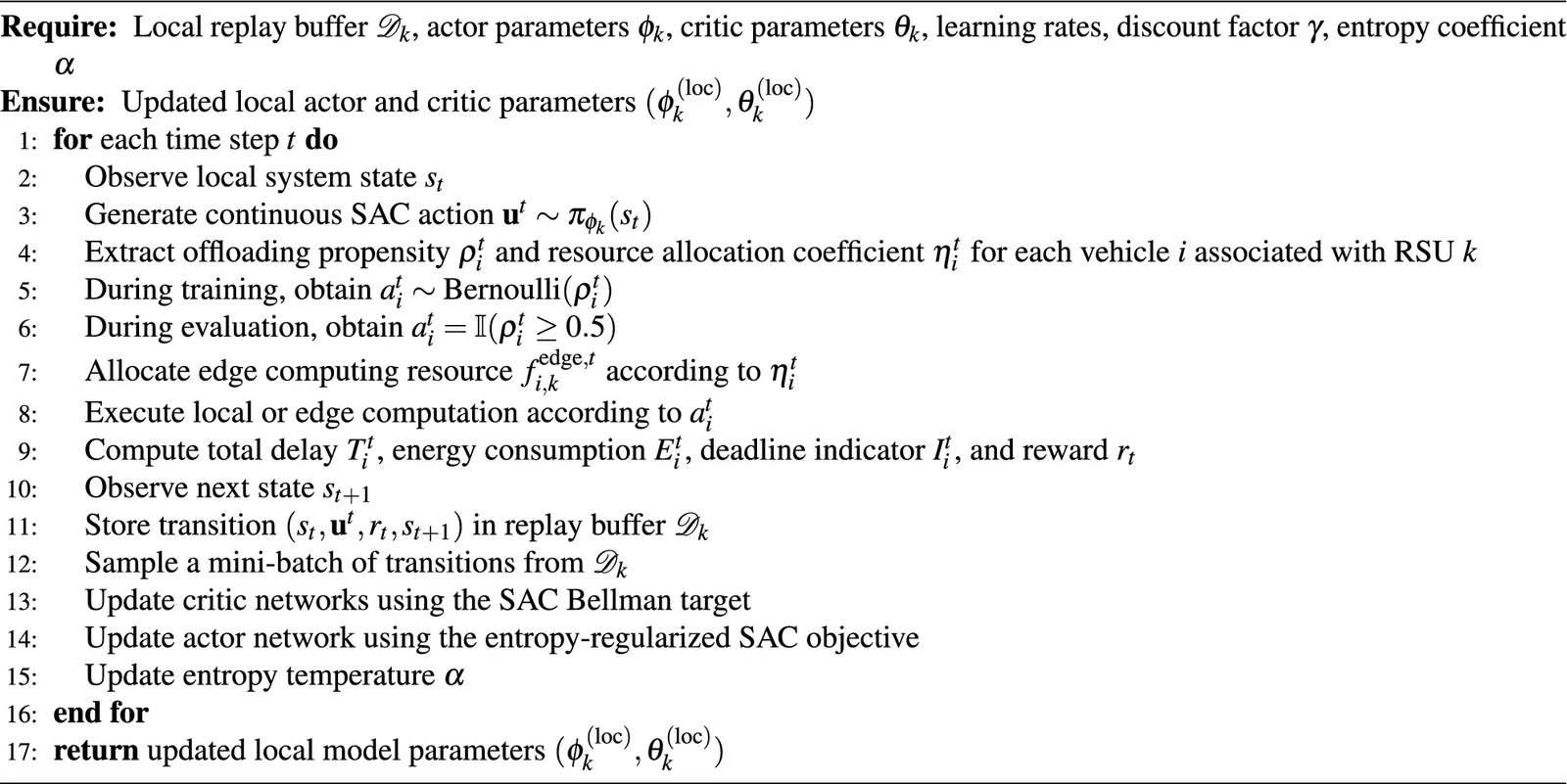
Local SAC-based hybrid action task offloading at RSU

To facilitate collaborative learning and reduce the coordinator’s exposure to raw vehicular data, RSUs do not hand over raw vehicle data to the coordinator. Rather, the participating RSUs periodically upload locally trained actor and critic parameters to the federated coordinator. Sample-size-weighted aggregation is used to update the global actor and critic parameters, as demonstrated in Eq. (38):

38 $$\begin{aligned} \phi ^{(g)} = \sum _{k=1}^{K} \frac{n_k}{n} \phi _k^{(\textrm{loc})}, \quad \theta ^{(g)} = \sum _{k=1}^{K} \frac{n_k}{n} \theta _k^{(\textrm{loc})}, \end{aligned}$$

where $n_k$ denotes the number of local training samples at RSU *k*, and $n=\sum _{k=1}^{K}n_k$ is the total number of samples across all participating RSUs. Because vehicular data are normally heterogeneous and non-independent and identically distributed (non-IID) by RSUs, a typical global aggregation can lead to reduced local adaptability. To solve this problem, a personal federated aggregation mechanism is adopted. After receiving the global model from the coordinator, each RSU forms a personalized actor and critic by combining the global parameters with its locally trained parameters, as defined in Eqs. (39) and (40):

39 $$\begin{aligned} \phi _k^{(p)} = \beta \phi ^{(g)} + (1-\beta )\phi _k^{(\textrm{loc})}, \end{aligned}$$

40 $$\begin{aligned} \theta _k^{(p)} = \beta \theta ^{(g)} + (1-\beta )\theta _k^{(\textrm{loc})}. \end{aligned}$$

Here, $\phi _k^{(p)}$ and $\theta _k^{(p)}$ denote the personalized actor and critic parameters of RSU *k*, respectively. The coefficient $\beta \in [0,1]$ controls the balance between global knowledge sharing and local RSU adaptation. When *β =0*, the RSU relies entirely on its local model, whereas *β =1* makes the RSU fully adopt the global model, which is equivalent to standard federated averaging without personalization. Intermediate values allow each RSU to combine global information with local specialization. In the main experiments, *β* is fixed at 0.50 based on the sensitivity analysis reported in Section "Performance evaluation"; therefore, it is treated as a fixed hyperparameter rather than an adaptively learned variable.

The personalized parameters $\phi _k^{(p)}$ and $\theta _k^{(p)}$ are used locally by RSU *k* for subsequent task offloading decisions and SAC training. They are not transmitted back to the coordinator separately. In the next communication round, each RSU uploads only the updated local model parameters obtained after local training from its personalized initialization. The federated aggregation and personalization workflow is summarized in Algorithm 2.

**Algorithm 2:**
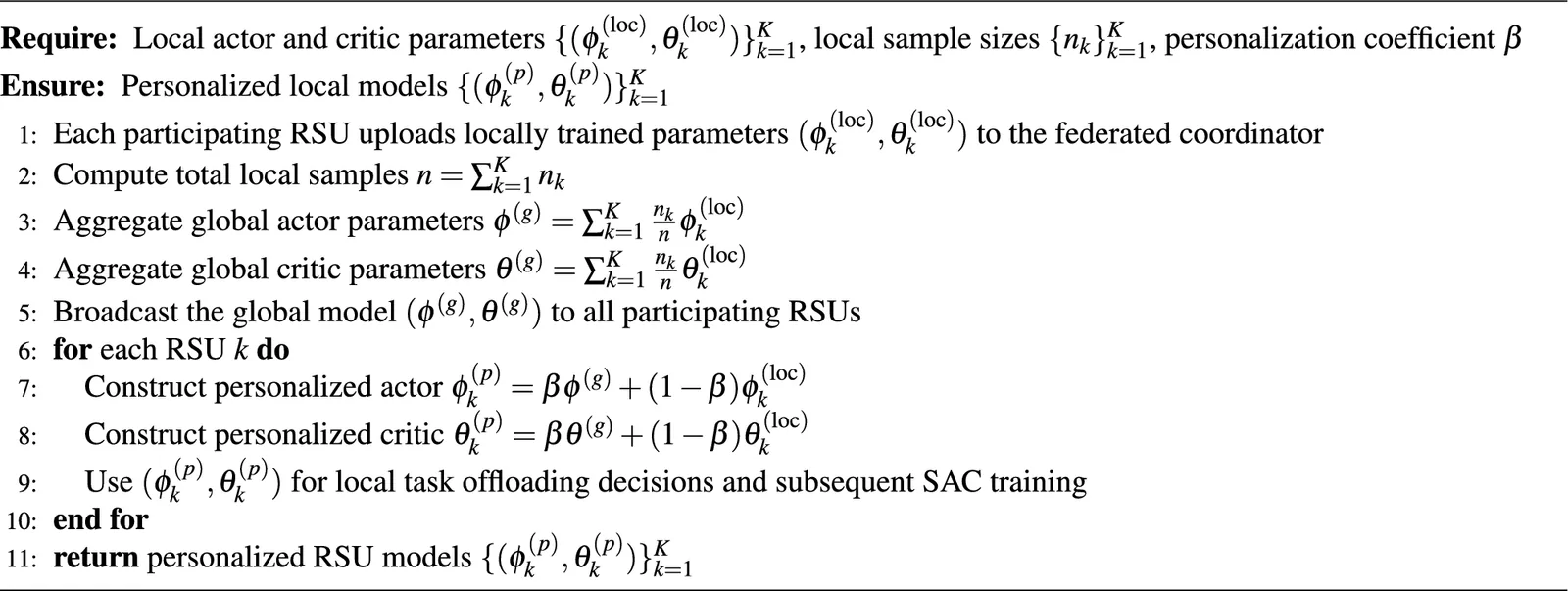
Federated aggregation and local personalization

Although SAC, FedAvg-style aggregation, deadline-aware reward shaping, and personalized model updates are individually established techniques, their joint use in the proposed framework changes the computational and communication structure of the offloading problem. A key analytical benefit of the proposed design is the conversion of the learning load from a centralized network into parallel updates of the RSUs and just static parameter aggregation from the coordinator. Let *|ϕ |* denote the number of actor-network parameters, *|θ |* denote the number of critic-network parameters, $B_s$ denote the mini-batch size, $E_{\textrm{loc}}$ denote the number of local training epochs, *G* denote the number of gradient-update steps in one communication round, and $K_p$ denote the number of participating RSUs in that round. Since SAC uses one actor and two critic networks, the effective SAC model size is defined in Eq. (41) as

41 $$\begin{aligned} P_{\textrm{SAC}} = |\phi | + 2|\theta |. \end{aligned}$$

Using Eq. (41), the local training complexity at one RSU in a communication round can be approximated as $\mathcal {O}(E_{\textrm{loc}}G B_s P_{\textrm{SAC}})$, because each update requires forward and backward propagation through the actor and critic networks. Across all participating RSUs, the total local computational cost per round becomes $\mathcal {O}(K_p E_{\textrm{loc}}G B_s P_{\textrm{SAC}})$. However, these local updates can be executed in parallel across RSUs; therefore, the wall-clock training cost is mainly governed by the slowest participating RSU rather than by the sum of all local computations. At the federated coordinator, aggregation requires weighted averaging of the received actor and critic parameters, giving a server-side complexity of $\mathcal {O}(K_p P_{\textrm{SAC}})$. Thus, the coordinator-side cost scales linearly with the number of participating RSUs and the model size, and does not scale directly with the number of raw vehicular observations generated within each RSU.

The communication cost is also different from centralized DRL training. If each model parameter is represented using *b* bits, the upload and download cost of one federated communication round is given in Eq. (42) as

42 $$\begin{aligned} C_{\textrm{FDRL}}^{\textrm{round}} = 2K_p P_{\textrm{SAC}} b, \end{aligned}$$

where the factor of two corresponds to uplink model upload and downlink global-model broadcast. For *R* federated rounds, the total parameter-exchange cost is given in Eq. (43) as

43 $$\begin{aligned} C_{\textrm{FDRL}}^{\textrm{total}} = 2RK_p P_{\textrm{SAC}} b. \end{aligned}$$

By contrast, centralized DRL requires the transmission of raw state, task, mobility, channel, and resource observations to a central learner. If RSU *k* produces $n_k$ local transitions per round and each transition has dimension $d_s$, the approximate centralized raw-data transfer per round is defined in Eq. (44) as

44 $$\begin{aligned} C_{\textrm{central}}^{\textrm{round}} = \sum _{k=1}^{K_p} n_k d_s b_s, \end{aligned}$$

where $b_s$ is the number of bits used to represent each state-transition element. Therefore, the proposed FDRL communication design becomes more efficient than centralized raw-data transmission when the condition in Eq. (45) is satisfied:

45 $$\begin{aligned} 2K_p P_{\textrm{SAC}} b < \sum _{k=1}^{K_p} n_k d_s b_s. \end{aligned}$$

This situation is expected when many vehicles are observed by the RSUs, when tasks are frequently generated, when good mobility information is provided, or when the channel and resource states are very high-dimensional. Thus, the communication benefit of the proposed framework is that repeated transfer of raw data is dropped and periodic transfer of models’ parameters is used instead. The downside is that RSUs perform more local computation, and the coordinator receives less raw vehicle data and performs lighter aggregation.

The non-IID nature of RSU-level data affects the behavior of FL because each RSU may observe different traffic density, vehicle mobility, task criticality, wireless channel quality, and available edge resources. Let $F_k(\omega )$ denote the expected local SAC objective at RSU *k*, where *ω* represents the combined actor–critic parameter vector. The global weighted objective is defined in Eq. (46) as

46 $$\begin{aligned} F(\omega )=\sum _{k=1}^{K_p}\frac{n_k}{n}F_k(\omega ), \end{aligned}$$

where $n=\sum _{k=1}^{K_p}n_k$ denotes the total number of local samples across the participating RSUs. A common way to express RSU-level heterogeneity is to measure the disagreement between local RSU optimization directions and the global federated direction. This heterogeneity measure is given in Eq. (47) as

47 $$\begin{aligned} \Delta _{\textrm{NIID}}(\omega )=\frac{1}{K_p}\sum _{k=1}^{K_p}\left\| \nabla F_k(\omega )-\nabla F(\omega )\right\| ^2. \end{aligned}$$

A larger $\Delta _{\textrm{NIID}}(\omega )$ indicates stronger disagreement between local RSU objectives and the global objective. Under such conditions, standard FedAvg may produce a global model that is acceptable on average but suboptimal for individual RSUs. The personalized aggregation used in this work partially reduces this effect by forming the personalized RSU model in Eq. (48) as

48 $$\begin{aligned} \omega _k^{(p)}=\beta \omega ^{(g)}+(1-\beta )\omega _k^{(\textrm{loc})}, \end{aligned}$$

where $\omega ^{(g)}$ is the global model, $\omega _k^{(\textrm{loc})}$ is the locally trained RSU model, and *β* controls the balance between global knowledge sharing and local specialization. This update does not eliminate non-IID drift, but it controls the extent to which each RSU follows the global model or preserves its local adaptation. In particular, for a local reference solution $\omega _k^\star$, the distance of the personalized model to the local reference can be bounded using Eq. (49) as

49 $$\begin{aligned} \left\| \omega _k^{(p)}-\omega _k^\star \right\| \le \beta \left\| \omega ^{(g)}-\omega _k^\star \right\| + (1-\beta )\left\| \omega _k^{(\textrm{loc})}-\omega _k^\star \right\| . \end{aligned}$$

Eq. (49) demonstrates that placing weight on the local (RSU-specific) model can help with local adaptation when the global model is very different from the local model due to non-IID heterogeneity in the data. However, if there are no significant differences in the RUS, a larger *β* can foster sharing knowledge across RUS. This provides an analytical explanation of the necessity of performing the personalization coefficient sensitivity analysis.

It is challenging to achieve a full closed-form convergence guarantee for the proposed framework since it fuses off-policy SAC, nonlinear function approximation, stochastic vehicular mobility, binary execution following continuous action generation, and non-IID federated updates. Hence, in this context, global optimality and formal convergence of the offloading policy to the optimal policy are not claimed. Combining the commonly used assumptions in federated stochastic optimization: bounded rewards, bounded gradient variance, Lipschitz continuous local objectives, and sufficiently small learning rates, the local updates from SAC can be read as approximate stochastic improvement steps toward stationary policies of the local RSU objectives. Then, federal averaging introduces a global-sharing step, and personalization reduces the mismatch between the global policy and the RSU’s local operating conditions. Here, convergence is defined as the stabilization of the training reward and system cost, rather than as proof of global optimality, all on an empirical basis. This restriction is clearly stated, and rigorous convergence bounds for federated off-policy reinforcement learning with non-IID vehicular dynamics are a direction for future research.

The proposed framework supports a FL environment in which raw vehicular data are never transferred to the federated coordinator; instead, only the model parameters are. In an attempt to give a quantitative privacy-related signal, the ratio of exposed raw data is defined in Eq. (50) as

50 $$\begin{aligned} \Gamma _{\textrm{raw}} = \frac{C_{\textrm{raw}}^{\textrm{method}}}{C_{\textrm{raw}}^{\textrm{centralized}}}, \end{aligned}$$

where $C_{\textrm{raw}}^{\textrm{method}}$ denotes the amount of raw vehicular data transmitted to the coordinator by a given method, and $C_{\textrm{raw}}^{\textrm{centralized}}$ denotes the raw data transmitted under centralized training. For centralized SAC, $\Gamma _{\textrm{raw}}=1$ because raw training observations are collected centrally. For the proposed FDRL framework, $\Gamma _{\textrm{raw}}=0$ under the assumed federated setting because RSUs transmit model parameters rather than raw vehicle trajectories, task records, channel observations, or mobility traces. This metric captures raw-data locality, not formal privacy against inference from model updates.

This design minimizes direct exposure of sensitive data like the vehicle location, task characteristics, mobility behavior, and observation of local channels. However, the presence of the model and model-parameter exchange should not be taken as an official guarantee of privacy. Even in adversarial conditions, model updates may leak information, such as via gradient leakage, model inversion attacks, or membership inference attacks. We make the following assumptions in this study: we have an honest-but-curious federated coordinator. The coordinator adheres to the aggregation protocol but may also attempt to deduce sensitive data by uploading model parameters. Within this premise, the suggested framework will minimize direct collection of raw data, as the coordinator will not be provided with local vehicular record data. However, the framework fails to ensure inference resistance against malicious use of cryptographic protection to infer model updates, or to prevent leakage of the same through deliberate manipulation or analysis of exchanged parameters.

The scope of privacy of the existing framework is thus limited to the locality of the raw data and minimized direct transmissions of data. Namely, RSUs transmit local SAC model parameters rather than raw vehicle trajectories, task records, channel observations, and mobility traces. This reduces the level of sensitive data made available to the coordinator relative at centralized DRL training, where raw data would have to be collected at a central server. However, this framework does not incorporate differential privacy noise injection, secure aggregation, homomorphic encryption, trusted execution environments, or adversarial privacy-attack testing. In this context, the term privacy-aware in this work means minimizing the exposure of raw data through federated training, not a mathematically guaranteed privacy mechanism. The combination of formal privacy defenses, including differentially private federated optimization, secure aggregation, and robustness analysis against model-inversion and membership-inference attacks, is regarded as a promising future direction.

The proposed FDRL model integrates distributed reinforcement learning with federation optimization to address the challenges of task offloading, scalability, and communication overhead, as well as the issue of data locality in vehicular edge computing. The framework facilitates adaptive task offloading in dynamic non-IID RSU-level operational regimes by integrating SAC-based hybrid action learning, personalized federated aggregation, and privacy-aware model sharing. The effectiveness of the proposed method is evaluated with the help of controlled simulations in the next section.

## Performance evaluation

In this section, the proposed FDRL framework is assessed by means of controlled simulation-based experiments in a vehicular edge computing system. The evaluation covers the simulation setup, baseline methods, non-IID data construction, convergence behavior, system cost, delay, energy consumption, deadline reliability, communication overhead, ablation analysis, personalization sensitivity, and robustness under RSU-level heterogeneity. All learning-based experiments were repeated 30 times with random seeds ranging from 1 to 30 to avoid relying on a single simulation trajectory. All reported numbers refer to the average of multiple independent runs unless otherwise indicated, and confidence is expressed in terms of standard deviation or 95% confidence intervals. The goal of this evaluation is not to assess realism in such city-scale deployments, but rather to conduct a controllable and repeatable comparison of centralized, federated, and personalized DRL-based offloading strategies under the same mobility, wireless, workload, and RSU-resource assumptions.

### Simulation setup

To test the proposed framework, we run controlled moderate-scale simulations, under dynamically arriving tasks and under vehicle motions, a variety of V2I channel conditions, limited RSU computing resources, and non-IID RSU-level data. The experimental setup is to analyze the behavior of a federated SAC-based task offloading system under controlled assumptions and should not be construed as a full-fledged deployment at a city scale.

The simulated road landscape is based on the topology of the Manhattan grid. The vehicles travel horizontally and vertically along road segments and adopt straight, left, or right movements at intersections with probabilities 0.6, 0.2, and 0.2, respectively. Vehicle velocity will be sampled using a bounded uniform distribution, $v_i^t \sim \mathcal {U}(v_{\min },v_{\max })$, and the vehicle positions will be updated at a time slot according to the current velocity and direction of the vehicle. The RSUs are placed at a fixed point on a roadway with a limited moment of coverage. A task that can be offloaded by a vehicle should be within the communication range of at least one RSU. Assuming there is a number of RSUs, the best-received-channel-condition criterion is used to select the serving RSU. In practice, on consecutive time slots, a handover event is triggered, and the handover delay in Eq. (12) is compared to the delay in executing the task.

For the wireless environment, the V2I link is modeled using distance-dependent path loss and small-scale fading. The channel gain between vehicle *i* and RSU *k* at time slot *t* is defined in Eq. (51) as

51 $$\begin{aligned} h_{i,k}^t = g_{i,k}^t \left( d_{i,k}^t\right) ^{-\alpha }, \end{aligned}$$

where $d_{i,k}^t$ is the distance between vehicle *i* and RSU *k*, *α* is the path-loss exponent, and $g_{i,k}^t$ is the small-scale fading gain. In the simulation, $g_{i,k}^t$ follows a Rayleigh fading model, and the achievable rate in Eq. (5) is used as a simplified Shannon-based approximation of the uplink V2I data rate. The wireless model assumes a noise-limited V2I environment in which resources are allocated orthogonally; therefore, co-channel interference, packet errors, channel estimation uncertainty, retransmission delay, and MAC-layer contention have not been explicitly modeled.

The simulated system contains *N=20*–50 vehicles and *K=4*–8 RSUs. Each RSU will have an edge server and supply vehicles within its communication range. Computation tasks are generated by a Poisson process, and their sizes are uniformly distributed with maximum and minimum sizes. The key system parameters, namely: mobility, wireless, handover, and task parameters, are summarized in Table 3, and the SAC and federated-learning hyperparameters are provided in Table 4.

To be able to compare the performance, both the conventional and the federated baselines are also incorporated, as can be seen in Table 5. In the traditional baselines, there are Local Execution, Edge Offloading, DQN-based offloading, DDPG-based offloading, and centralized SAC (C-SAC). To give a stronger comparison with the recent studies on federated RL offloading, we also include FedAvg-DQN, FedAvg-DDPG, FedAvg-SAC, and Fed-MADRL. The baselines are that the proposed framework can be compared to definite offloading strategies, centralized DRL frameworks, and federated RL options within the same system model, mobility setting, wireless assumption, task-arrival process, and RSU resource constraint. Recently, some federated RL offloading approaches are based on scenario-specific components, such as UAV-assisted low-altitude fog, hierarchical regional controller, contextual clustering, game-theoretic resource competition, or unavailable implementation details. Thus, it is challenging to precisely reproduce all newer techniques without having the source code, hyperparameters, and the exact simulation environments. To this end, the proposed approach is contrasted with reproducible algorithmic equivalents, within a common evaluation framework. Their results are expected to convey the message about the performance of the experimental conditions under the combined controlled assumptions.

Lastly, the simulation simplifies a number of physical-layer and traffic-level specifics. Specifically, these lack realistic microscopic traffic traces, co-channel interference, packet-level transmission errors, channel-estimation uncertainty, retransmission, and MAC-layer contention. Further work on a more realistic V2X assessment of city-scale traffic traces and on wireless interference modeling is left to other studies.

**Table 3:** System, mobility, wireless, handover, and task simulation parameters used in the experiments.

Parameter	Symbol	Value
Number of vehicles	*N*	20–50
Number of RSUs	*K*	4–8
Vehicle speed range	$[v_{\min }, v_{\max }]$	30–80 km/h
Simulation time slot	*Δ t*	1 s
RSU coverage radius	$r_{\textrm{RSU}}$	300 m
Straight movement probability	$p_s$	0.6
Left-turn probability	$p_l$	0.2
Right-turn probability	$p_r$	0.2
Channel bandwidth	*B*	10 MHz
Transmission power	$P_i$	0.5 W
Noise power	$\sigma ^2$	$10^{-9}$ W
Path-loss exponent	*α*	3
Small-scale fading	$g_{i,k}^t$	Rayleigh fading
Backhaul bandwidth	$B_{\textrm{bh}}$	20 MHz
Backhaul transmit power	$P_{\textrm{bh}}$	1 W
Backhaul noise power	$\sigma _{\textrm{bh}}^2$	$10^{-9}$ W
Signaling-message size	$S_{\textrm{sig}}$	0.05–0.10 MB
Service-context size	$S_{\textrm{ctx}}$	0.5–2 MB
Processing/authentication delay	$T_i^{\textrm{proc},t}$	2–5 ms
Average handover delay coefficient	*δ*	5–20 ms
Vehicle CPU frequency	$f_i^{\textrm{loc}}$	1–2 GHz
Maximum RSU CPU frequency	$f_k^{\max }$	5–10 GHz
Energy coefficient	*κ*	$10^{-27}$
Task size	$D_i^t$	0.5–3 MB
CPU cycles per bit	$c_i^t$	500–1000 cycles/bit
Task criticality class	$\chi _i^t$	Critical, delay-sensitive, best-effort
Deadline-violation tolerance	*\varepsilon*	0.05–0.15
Energy weight	*λ*	0.1
Deadline penalty weights	$\mu _{\chi _i^t}$	5, 2, 1 for critical, delay-sensitive, best-effort

**Table 4:** Hyperparameters and federated-learning settings used in the experiments.

Parameter	Value
Actor learning rate	0.0003
Critic learning rate	0.0003
Discount factor (*γ*)	0.99
Replay buffer size	100,000
Batch size	64
Target update rate (*τ*)	0.005
Entropy coefficient (*α*)	0.2
Federated aggregation interval	10 episodes
Local training epochs	5
Maximum number of RSUs	8
Participating RSUs per round	4
Client participation ratio	50%
Personalization coefficient (*β*)	0.50
Independent runs	30
Random seeds	1–30
Parameter precision	32-bit floating point
Compared federated baselines	FedAvg-DQN, FedAvg-DDPG, FedAvg-SAC, Fed-MADRL

**Table 5:** Baseline methods used for performance comparison.

Baseline	Description
Local	All computation tasks are executed locally on vehicles without edge offloading.
Edge	Tasks are offloaded to the associated RSU whenever the vehicle is within RSU coverage.
DQN	A value-based DRL offloading method using discrete binary offloading decisions.
DDPG	A deterministic actor-critic DRL method for continuous offloading/resource-control decisions.
C-SAC	Centralized SAC trained using centrally collected system observations.
FedAvg-DQN	RSUs train local DQN agents and aggregate model parameters using federated averaging.
FedAvg-DDPG	RSUs train local DDPG agents and aggregate actor-critic parameters using federated averaging.
FedAvg-SAC	RSUs train local SAC agents and aggregate model parameters using standard FedAvg without personalization.
Fed-MADRL	Federated multi-agent DRL offloading baseline adapted from recent federated RL vehicular offloading studies.
Proposed FDRL	Federated SAC with hybrid offloading/resource action, deadline-aware reward, handover modeling, and personalized aggregation.

### Fairness of baseline implementation

The same task-arrival process, mobility model, RSU deployment, wireless-channel model, and computation-resource constraints were used in each instance of implementing a learning-based baseline. In the case of centralized baselines, it was assumed that the learning agent could gather system-level observations during training. In the federated baselines, each RSU was trained with its local model, observing only local transitions and uploading monthly to the federated coordinator. The aggregation interval, number of local training epochs (per batch), size of replay buffer, batch size, and the learning rates were kept the same across methods when algorithmically applicable. The methods adopted as baseline to compare performance can be summarized in Table 5.

In FedAvg-DQN, FedAvg-DDPG, and FedAvg-SAC, the local network structure is exactly the same as that of the centralized algorithmic variant, and the model parameters are averaged using federated averaging based on the size of the samples each client has collected. FedAvg-SAC is not exactly the same as the proposed FDRL framework, which jointly introduces a criticality-aware deadline penalty and a handover-aware reward formulation, and FedAvg training involves no personalized aggregation. Fed-MADRL is implemented as a federated multi-agent DRL baseline to learn a local offloading policy for each RSU agent, with the coordinator periodically sharing model parameters among agents. The implementation choices enable centralized, federated, and personalized reinforcement-learning methods to be compared within the same simulation framework.

### Non-IID data construction

In order to determine statistical heterogeneity at the RSU-level, local training data that are never IID are built across different RSUs. Various RSUs can monitor traffic rates, task arrival rates, vehicle speeds, task criticality patterns, and wireless channel characteristics. Hence, it is unrealistic to assume that the data distributions across all RSUs are the same.

In the simulation, each RSU *k* is assigned a heterogeneous local environment characterized by its task arrival rate $\lambda _k^{\textrm{task}}$, task criticality distribution $p_k(\chi )$, vehicle density, and average channel quality. The task criticality distribution at RSU *k* is sampled from a Dirichlet distribution, as defined in Eq. (52):

52 $$\begin{aligned} \textbf{p}_k = \left[ p_k(\textrm{critical}), p_k(\mathrm {delay\text {-}sensitive}), p_k(\mathrm {best\text {-}effort}) \right] \sim \textrm{Dirichlet} \left( \alpha _{\textrm{het}}\textbf{p}_0 \right) , \end{aligned}$$

where $\textbf{p}_0$ denotes the global task criticality distribution and $\alpha _{\textrm{het}}$ controls the degree of RSU-level statistical heterogeneity. A larger $\alpha _{\textrm{het}}$ produces more similar task criticality distributions across RSUs, while a smaller value produces stronger task-distribution skew and stronger non-IID behavior.

Besides the skew of criticality of tasks, heterogeneity is added with the workload on the channel and RSU-specific workload. The local rate of task arrival at RSU *k* is sampled around the global average rate, and the vehicle density and channel quality are varied across RSUs to represent spatially heterogeneous vehicular conditions. In the experiments, quantitative non-IID settings were utilized; they are summarized in Table 6. We use $\alpha _{\textrm{het}}=10$ for mild non-IID, $\alpha _{\textrm{het}}=1$ for moderate non-IID, and $\alpha _{\textrm{het}}=0.3$ for high non-IID. The moderate non-IID setting is used as the default configuration unless otherwise stated.

**Table 6:** Quantitative non-IID settings used for RSU-level heterogeneity analysis.

Non-IID level	Dirichlet parameter $\boldsymbol{\alpha _{\textrm{het}}}$	Task distribution skew	Task arrival variation	Description
IID / near-IID	$\alpha _{\textrm{het}} \rightarrow \infty$	None or negligible	Same across RSUs	Nearly identical RSU-level task and workload distributions
Mild non-IID	10	Low	$\pm 10\%$ around global mean	Similar task distributions with small workload variation
Moderate non-IID	1	Medium	$\pm 25\%$ around global mean	Moderate differences in task criticality, workload, and channel quality
High non-IID	0.3	High	$\pm 50\%$ around global mean	Strongly skewed task distributions and heterogeneous local environments

### Reproducibility and statistical reporting

To account for the stochastic nature of reinforcement learning and vehicular simulation, each learning-based experiment was repeated over $N_{\textrm{run}}=30$ independent runs using random seeds 1–30. These seeds control vehicle initialization, mobility decisions at intersections, task-arrival events, task-size sampling, task-criticality assignment, Rayleigh fading samples, neural-network initialization, replay-buffer sampling, mini-batch selection, and exploration noise. Unless otherwise stated, reported results represent the mean value across the independent runs. For a performance metric *x*, the sample mean is computed using Eq. 53 as

53 $$\begin{aligned} \bar{x}=\frac{1}{N_{\textrm{run}}}\sum _{r=1}^{N_{\textrm{run}}}x_r , \end{aligned}$$

where $x_r$ denotes the value obtained in the *r*th independent run. The corresponding sample standard deviation is computed using Eq. 54 as

54 $$\begin{aligned} s_x= \sqrt{ \frac{1}{N_{\textrm{run}}-1} \sum _{r=1}^{N_{\textrm{run}}} (x_r-\bar{x})^2 }. \end{aligned}$$

The 95% confidence interval is then defined in Eq. 55 as

55 $$\begin{aligned} \textrm{CI}_{95\%} = \bar{x} \pm t_{0.975,N_{\textrm{run}}-1} \frac{s_x}{\sqrt{N_{\textrm{run}}}}, \end{aligned}$$

where $t_{0.975,N_{\textrm{run}}-1}$ is the critical value of the Student’s *t* distribution with $N_{\textrm{run}}-1$ degrees of freedom. The use of the Student’s *t* distribution is preferred over a fixed normal approximation because the number of independent runs is finite. In convergence plots, the displayed curve represents the mean reward across 30 runs, while the shaded region represents the 95% confidence interval. Where moving-average smoothing is used for readability, the confidence interval is computed from the run-level values before smoothing so that uncertainty is not artificially reduced.

For the purpose of further support for statistical interpretation, paired comparisons were performed among the same random seeds. The main baseline methods to compare were centralized SAC, the best centralized method, and FedAvg-SAC, the closest baseline without personalization among federated SAC-based methods. A two-sided paired *t*-test was used for normally distributed paired differences. When the paired differences did not satisfy the assumption of normality, the Wilcoxon signed-rank test was used instead. The *p<0.05* threshold was used as the significance level. When more than one metric was computed, the Holm-Bonferroni correction was performed to minimize false positive findings.

Thus, in addition to mean performance, the run-to-run variability is taken into account in the interpretation of the results. In particular, numeric differences are interpreted with caution in the absence of significance in the paired statistical test, when the confidence intervals overlap, or when the numerical differences are not substantially different from the centralized SAC. This is significant because obtaining system-level observations during training would make centralized SAC available and, therefore, make it a solid baseline. The goal of the proposed FDRL framework is not to drastically outperform centralized SAC in all aspects, but to partially achieve competitive or better performance, reduce raw data transmission in the centralized system, and implement FL on the RSUs’ side.

All the compared methods were tested using the same mobility and workload, wireless channel, and resources available at the RSUs to ensure reproducibility. The controlled Manhattan-grid mobility model is used for the simulation. The cars are generated on horizontal and vertical road sections and are assigned probabilities of 0.6, 0.2, and 0.2 for making straight, left, and right movements at intersections, respectively. The values of vehicle speeds, RSU coverages, wireless bandwidth, task sizes, CPU-cycle requirements, and deadline-related parameters are the same as those given in Table 3. The arrival of tasks is modeled as a Poisson process, and non-IID task criticality and workload heterogeneity are created at the RSU level. The same random seed setting is used across baselines for paired and fair comparisons. Table 7 gives a summary of the performed reproducibility and fairness measurements, which have been used to ensure identical mobility, workload, wireless channel, and task-generation, as well as identical resources offered by the RSU when comparing all the methods.

**Table 7:** Reproducibility and fairness controls used in the simulation experiments.

Component	Reproducibility control
Mobility model	A controlled Manhattan-grid topology was used. Vehicles moved along horizontal and vertical road segments with fixed straight, left-turn, and right-turn probabilities of 0.6, 0.2, and 0.2, respectively.
RSU association and handover	Vehicles were associated with the RSU providing the best received V2I channel condition within coverage. A handover was triggered when the serving RSU changed between consecutive time slots.
Task generation	Task arrivals followed a Poisson process. Task size, CPU-cycle demand, deadline tolerance, and criticality class were generated according to the ranges and settings reported in Table ??.
Wireless channel generation	V2I channels used distance-dependent path loss and Rayleigh fading. The same channel-generation procedure was applied to all compared methods.
Independent runs	Each learning-based experiment was repeated over 30 independent runs using random seeds 1–30.
Seed control	The seeds controlled vehicle initialization, mobility decisions, task arrivals, task sizes, task criticality, fading samples, neural-network initialization, replay-buffer sampling, and mini-batch selection.
Baseline fairness	All baselines were evaluated using the same mobility traces, workload seeds, wireless-channel seeds, RSU-resource constraints, and task-generation settings.
Statistical reporting	Results were reported as mean values with standard deviation or 95% confidence intervals. Paired statistical comparisons were performed against C-SAC and FedAvg-SAC.

### Communication-cost calculation

Communication overhead is determined to be the total amount of data transferred in training. In the centralized SAC, the central learner receives training observations from the involved RSUs. The cumulative communication cost after *R* communication rounds is defined in Eq. (56) as

56 $$\begin{aligned} C_{\mathrm {C\text {-}SAC}}(R) = C_{\textrm{init}}^{\textrm{cen}} + \sum _{r=1}^{R} m_r S_{\textrm{data}}, \end{aligned}$$

where $C_{\textrm{init}}^{\textrm{cen}}$ denotes the initialization and synchronization overhead, $m_r$ is the number of participating RSUs in round *r*, and $S_{\textrm{data}}$ is the average amount of raw training data transmitted by each participating RSU in one round. Therefore, the cost of centralized communication primarily goes into conveying training observations to the overall learner. In federated methods, RSUs transmit local model parameters and get the aggregated global model at the end of each communication round. Thus, the federated communication cost will consist of uplink model upload and downlink global-model broadcast as provided in Eq. (57):

57 $$\begin{aligned} C_{\textrm{FL}}(R) = C_{\textrm{init}}^{\textrm{FL}} + \sum _{r=1}^{R} m_r \left( S_{\textrm{up}}+S_{\textrm{down}} \right) , \end{aligned}$$

where $C_{\textrm{init}}^{\textrm{FL}}$ denotes the initial model synchronization overhead, $S_{\textrm{up}}$ is the uploaded local model-update size, and $S_{\textrm{down}}$ is the downloaded global-model size. No model compression, pruning, sparsification, or quantization is applied in the current implementation. All model parameters are transmitted using 32-bit floating-point precision, so the transmitted model size is calculated in Eq. (58) as

58 $$\begin{aligned} S_{\theta } = \frac{32 n_{\theta }}{8 \times 10^{6}}, \end{aligned}$$

where $n_{\theta }$ denotes the number of transmitted model parameters and $S_{\theta }$ is measured in MB. When the uploaded and downloaded model sizes are assumed to be the same, Eq. (57) is simplified as

59 $$\begin{aligned} C_{\textrm{FL}}(R) = C_{\textrm{init}}^{\textrm{FL}} + 2 \sum _{r=1}^{R} m_r S_{\theta }. \end{aligned}$$

In the experiments, communication overhead is reported over *R=50* communication rounds, using the parameter settings summarized in Table 8. The number of RSUs per round is *K=8* and $m_r=4$, meaning that RSUs participate in every round, corresponding to a client participation proportion of 50%. The number of episodes between which aggregation should occur is set at 10. Table 14 gives the values on communication cost, which are obtained as the following equations: (56)–(59). By which the calculations explicitly cover both uplink and downlink transmission in federated approaches, and use an uncompressed 32-bit model parameter.

**Table 8:** Communication-cost parameters used for centralized and federated training.

Parameter	Symbol	Value
Communication rounds	*R*	50
Maximum number of RSUs	*K*	8
Participating RSUs per round	$m_r$	4
Client participation ratio	$m_r/K$	50%
Aggregation interval	–	10 episodes
Parameter precision	*b*	32 bits floating point
Model compression/quantization	–	Not applied
Centralized raw-data transfer per RSU per round	$S_{\textrm{data}}$	1.875 MB
C-SAC initialization/synchronization overhead	$C_{\textrm{init}}^{\textrm{cen}}$	125 MB
FedAvg-DDPG model-update size	$S_{\theta }$	0.250 MB
FedAvg-SAC model-update size	$S_{\theta }$	0.219 MB
Fed-MADRL model-update size	$S_{\theta }$	0.250 MB
Proposed FDRL model-update size	$S_{\theta }$	0.188 MB
FDRL initialization/synchronization overhead	$C_{\textrm{init}}^{\textrm{FL}}$	45 MB
Federated transmission direction	–	Uplink model upload and downlink global-model broadcast

### Convergence performance

**Fig. 2:**
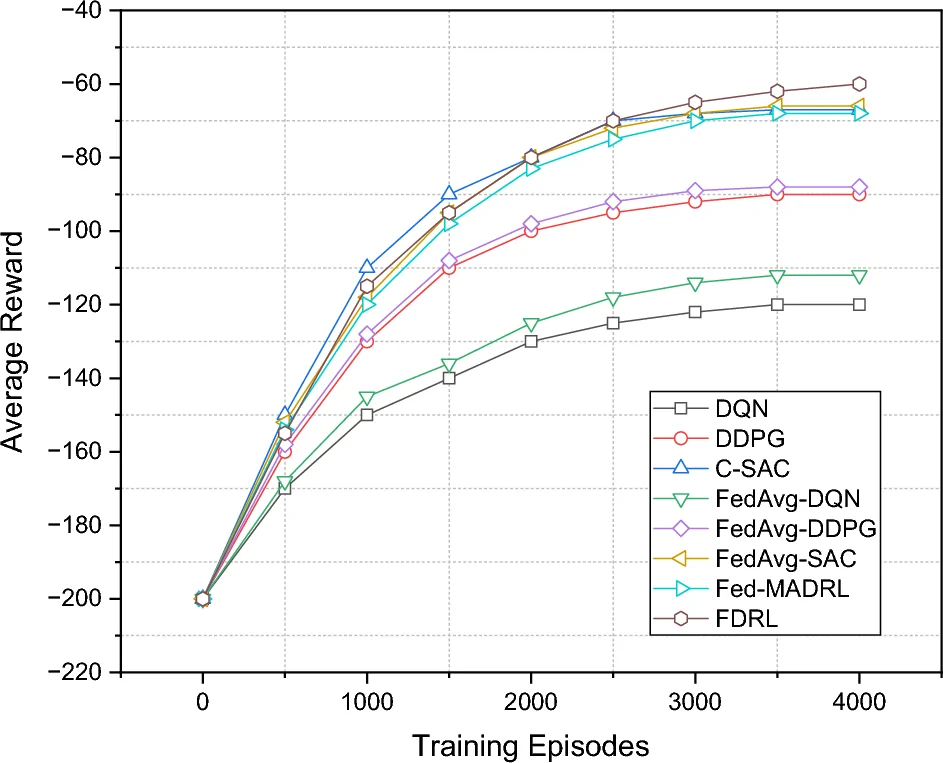
Convergence of average reward over training episodes for centralized and federated baselines. The curves represent the mean over 30 independent runs, and the shaded regions represent 95% confidence intervals.

We initially compare the convergence behavior of the proposed FDRL framework with the centralized and FL-based baselines, as demonstrated in Fig. 2 and Table 9. They are compared with DQN, DDPG, centralized SAC (C-SAC), FedAvg-DQN, FedAvg-DDPG, FedAvg-SAC, Fed-MADRL, and the proposed FDRL framework. Federated baselines should be included to determine whether the proposed design provides value addition over traditional centralized DRL approaches.

DQN is sensitive to value-based learning and experiences larger fluctuations in rewards due to its slower convergence and larger fluctuations in the value learning. DDPG is also more robust to convergence than DQN, but its deterministic policy is susceptible to exploration noise and non-stationary vehicular behaviors. The inference speed of C-SAC is higher than these baselines because, in C-SAC, the rate of inference improvement is precisely the gradient difference; however, in these baselines, the gradient difference is only approximated by the gradient difference. The federated baselines minimize the transmission of raw data, since the RSU uses model parameters to train vehicles rather than vehicle observations. However, FedAvg-DQN and FedAvg-DDPG inherit the shortcomings of their underlying learners, whereas FedAvg-SAC does not personalize non-IID RSU-level data. Fed-MADRL enhances multi-agent non-stationarity, and heterogeneous local observations explain why convergence in fed-MADRL remains compromised.

The proposed FDRL framework exhibits stable convergence and achieves the highest final reward among the compared methods. Even though C-SAC converges after fewer episodes, this does not indicate overall superiority, as it relies on centralized training. By comparison, FDRL involves local training of RSUs and federal synchronization, which incurs a slight delay in convergence but achieves better steady-state rewards, lower communication overhead, and reduced raw-data transmission. Therefore, the convergence obtained with FDRL indicates a sensible trade-off: C-SAC converges to a centralized performance result much faster, whereas FDRL converges to a centralized performance result at a much more slowly, neglecting raw-data locality in the FL framework.

**Table 9:** Convergence speed and final reward comparison with centralized and federated baselines. C-SAC converges faster due to centralized training, whereas the proposed FDRL achieves a higher final reward under federated training.

Algorithm	Episodes to converge	Final reward
DQN	4500	− 120
DDPG	3500	− 95
C-SAC	2500	− 70
FedAvg-DQN	4200	− 112
FedAvg-DDPG	3300	− 88
FedAvg-SAC	3000	− 66
Fed-MADRL	3100	− 68
Proposed FDRL	2800	− 60

**Fig. 3:**
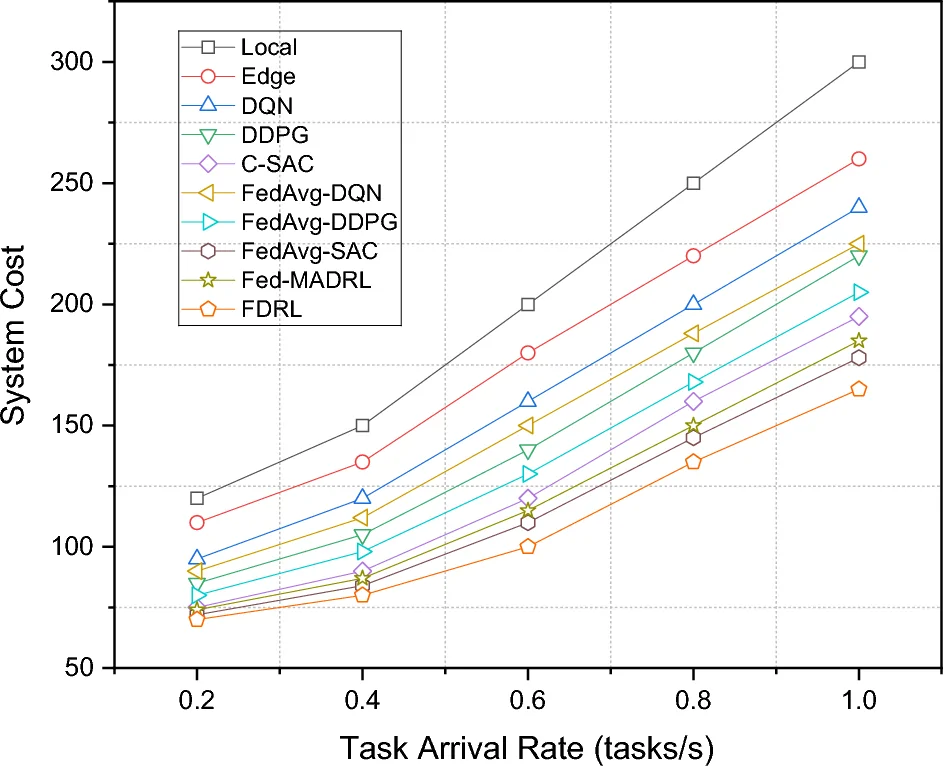
System cost under varying task arrival rates for centralized and federated baselines. Results are reported as the mean over 30 independent runs.

### System cost analysis

We then consider the total cost of the system at varied task arrival rates as illustrated in Fig. 3 and Table 10. The system cost is the sum of the task execution delay, energy consumption, and the penalty for defaulting on the deadline, enabling the overall efficiency of each offloading strategy to be compared under varying-intensity workloads.

The cost of the system increases with all the methods; the faster the task arrival rate, the higher the system cost up to a limit where the system cost levels off. The Local baseline lacks onboard computation units, and the Edge baseline is also inefficient due to high task density, as it aggressively offloads without adaptive resource-aware control. DQN and DDPG are less expensive than fixed offloading strategies, but their performance is constrained by instability in learning under dynamic vehicle conditions. With the federated baselines, there is an overall improvement over the non-federated counterparts, as the RSU agents learn local observations while still benefiting from model aggregation. Non-IID RSU-level data is not adequately addressed by FedAvg-SAC, which uses standard global averaging to construct it. Fed-MADRL enhances distributed coordination, even in the presence of heterogeneous local observations and multi-agent non-stationarity.

The suggested FDRL framework minimizes the system cost in response to the task arrival rates over the considered time period. This has been enhanced largely through the use of SAC-based hybrid action learning, adaptive offloading and resource allocation, deadline-criticality-aware penalty design, and personalized federated aggregation. Unlike C-SAC, the proposed method avoids centralized collection of raw data, and unlike standard FedAvg-SAC, it preserves local RSU adaptation while still benefiting from global knowledge sharing.

**Table 10:** Average system cost under different workloads for centralized and federated baselines.

Arrival rate	Local	Edge	DQN	DDPG	C-SAC	FedAvg-DQN	FedAvg-DDPG	FedAvg-SAC	Fed-MADRL	FDRL
0.2	120	110	95	85	75	90	80	72	74	70
0.5	180	160	140	125	110	132	118	102	105	95
0.8	250	220	200	180	160	188	168	145	150	135

### Task delay performance

**Fig. 4:**
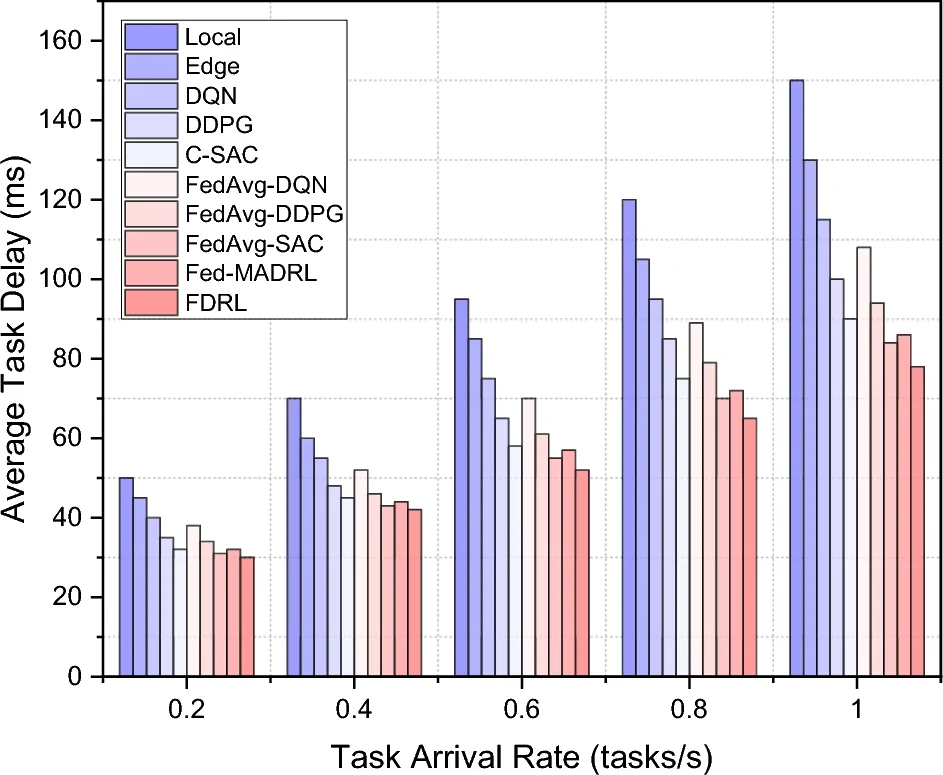
Average task delay under different workloads for centralized and federated baselines. Results are reported as the mean over 30 independent runs.

The average task delay with varying task arrival rate is presented in Fig. 4 and Table 11. Task delay is a key metric in vehicular edge computing, as timely execution is necessary for cooperative perception, collision warning, and navigation assistance applications. Since the rate at which tasks arrive also increases, the average delay time obtained using all methods increases because the computational demand is higher and RSUs contend for resources more often with a higher task arrival rate.

The Local baseline is capable of providing reasonable delay at low rates of task arrival since the tasks are processed, but no transmission overhead is incurred. But at higher workloads, local execution becomes inefficient due to limited onboard computing resources. The Edge baseline alleviates load on local devices but can experience transmission delay and congestion at the RSU due to offloading without resource-aware adaptive control. DQN and DDPG have better delay performance than fixed strategies, but their learning stability is affected by channel and workload variations, as well as mobility. The federated baselines enhance distributed adaptation by enabling RSUs to learn local policies and share model parameters. FedAvg-DQN and FedAvg-DDPG achieve moderate delay reduction, whereas FedAvg-SAC benefits from entropy-regularized exploration and continuous control. However, ordinary FedAvg-SAC does not necessarily adapt to heterogeneous RSU-level traffic and channel distributions due to the lack of personalization. Fed-MADRL is an improved approach to distributed decision-making, but its operation also fails in non-steady multi-agent interactions.

The proposed FDRL framework has the lowest average delay and lower worst-case delay than the baselines considered. This is significantly contributed to by the hybrid action design, which simultaneously factors in offloading propensity and RSU resource allocation, coupled with the handover-aware delay model, and personalized federated aggregation. The reduced worst-case delay indicates greater robustness under heavier workloads and on-the-fly mobility-driven RSU reassociation, which is particularly important for delay-sensitive and safety-critical vehicular applications.

**Table 11:** Average and worst-case task delay comparison for centralized and federated baselines.

Algorithm	Avg delay (ms)	Max delay (ms)
Local	85	210
Edge	75	190
DQN	65	160
DDPG	55	140
C-SAC	48	120
FedAvg-DQN	61	150
FedAvg-DDPG	52	132
FedAvg-SAC	47	115
Fed-MADRL	49	118
FDRL	45	105

**Fig. 5:**
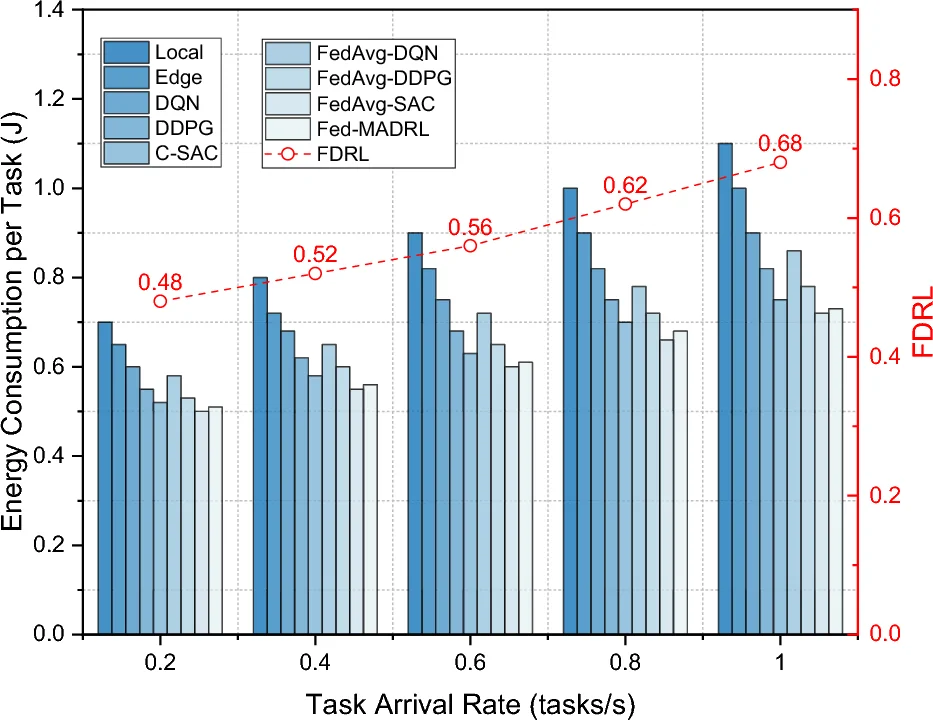
Energy consumption per task under varying workloads for centralized and federated baselines. Results are reported as the mean over 30 independent runs.

### Energy consumption analysis

The values of average energy consumed per task at different task arrival rates are depicted in Fig. 5 and Table 12. Energy security is significant for vehicular edge computing, as vehicles have limited onboard energy access, whilst excess offloading can increase wireless transmission energy consumption. Thus, to be effective, an offloading policy has to scale local computation energy and transmission-related energy cost.

The Local baseline has a greater energy consumption since all the computation tasks are done using onboard vehicle resources, which is not so efficient for tasks with a high compute requirement. The edge baseline reduces onboard computation pressure but increases energy consumption under heavier workloads due to extensive wireless transmission and execution on the RSU side. DQN and DDPG incur lower energy consumption than fixed strategies because they learn to adapt to offloading. Adaptation is more distributed across the federated baselines than in non-federated methods. Each of FedAvg-DQN and FedAvg-DDPG can safely have RSUs learn local policies and share model parameters, but cannot achieve state-of-the-art performance due to limitations in their underlying learners. Under non-IID RSU-level workload, traffic, and channel conditions, FedAvg-SAC consumes less energy by enabling smoother continuous control, but conventional global aggregation can result in a loss of adaptability. In addition to distributed cooperation, Fed-MADRL is prone to instability in the performance of multi-agent learning for energy efficiency.

The proposed FDRL framework has minimized average energy consumption among the thought-about baselines. This is enhanced by the hybrid action representation, in which the SAC actor learns not only the offloading propensity but also the locations of edges and the allocation of resources. The proposed method thus avoids unnecessary local heavy computation and over-offloading when transmission energy is expensive. Federated aggregation with personalization further enhances energy efficiency because each RSU can adapt to the local vehicular environment while still sharing ideas with other users worldwide.

**Table 12:** Average energy consumption per task for centralized and federated baselines.

Algorithm	Energy per task (J)
Local	0.95
Edge	0.85
DQN	0.78
DDPG	0.70
C-SAC	0.65
FedAvg-DQN	0.74
FedAvg-DDPG	0.67
FedAvg-SAC	0.61
Fed-MADRL	0.63
FDRL	0.58

### Deadline violation and reliability

**Fig. 6:**
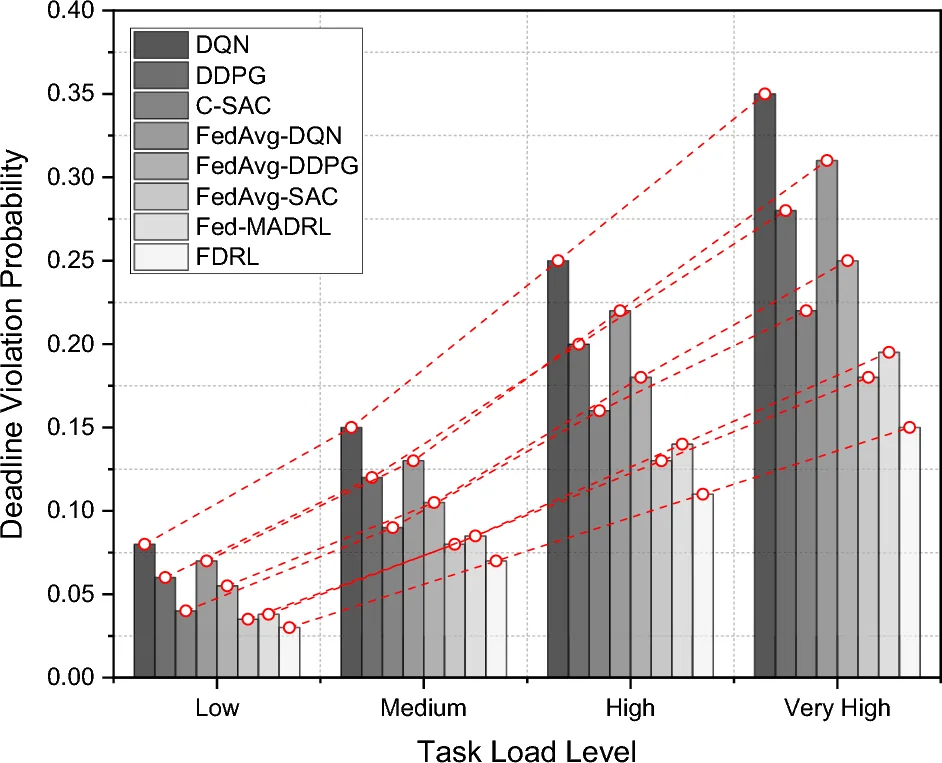
Deadline violation probability under different system loads for centralized and federated baselines. Results are reported as the mean over 30 independent runs.

In order to determine reliability, we test probabilities of violation deadline at various sense levels of task load, as shown in Fig. 6 and Table 13. This measure is significant for safety-sensitive vehicular use, as a work outcome might become impractical if executed after its timeframe.

The probability of deadline violation of all methods increases as the task load becomes heavier, due to which the RSU resource contention, transmission demand, and task waiting time increase accordingly. DQN and DDPG are less reliable in high-load scenarios because their policies do not explicitly account for task criticality and deadline risk. Entropy-regularized exploration and non-evolving policy learning enable C-SAC to outperform DQN and DDPG, but it requires centralized access to system-level observations. The federated baselines enhance distributed learning and minimize the direct transmission of raw data. FedAvg-DQN and FedAvg-DDPG show a moderate improvement in reliability, with FedAvg-SAC reported to have a more stable policy-learning process. Non-IID RSU-level workload and traffic patterns are, however, not completely addressed by standard federated averaging. The Fed-MADRL facilitates multi-agent coordination, though increased deadlines may still occur under heterogeneous local observations or high-frequency handovers due to mobility.

The proposed FDRL algorithm has the lowest probability of violation of deadline under the three loads of tasks considered. The main reasons for this improvement are the criticality-dependent penalty term $\mu _{\chi _i^t}$, probabilistic deadline modeling, and the formulation of delay based on handover considerations. The proposed solution, by imposing harsher penalties for failure to meet deadlines on critical workloads, will urge the agent to prioritize safety-sensitive workloads when computing and communication resources are scarce, leading to higher reliability for delay-sensitive vehicular applications.

**Table 13:** Deadline violation probability under different task loads for centralized and federated baselines.

Task load	DQN	DDPG	C-SAC	FedAvg-DQN	FedAvg-DDPG	FedAvg-SAC	Fed-MADRL	FDRL
Low	0.08	0.06	0.04	0.07	0.055	0.035	0.038	0.03
Medium	0.15	0.12	0.09	0.13	0.105	0.080	0.085	0.07
High	0.25	0.20	0.16	0.22	0.180	0.130	0.140	0.11
Very High	0.35	0.28	0.22	0.31	0.250	0.180	0.195	0.15

### Communication overhead and privacy scope

**Fig. 7:**
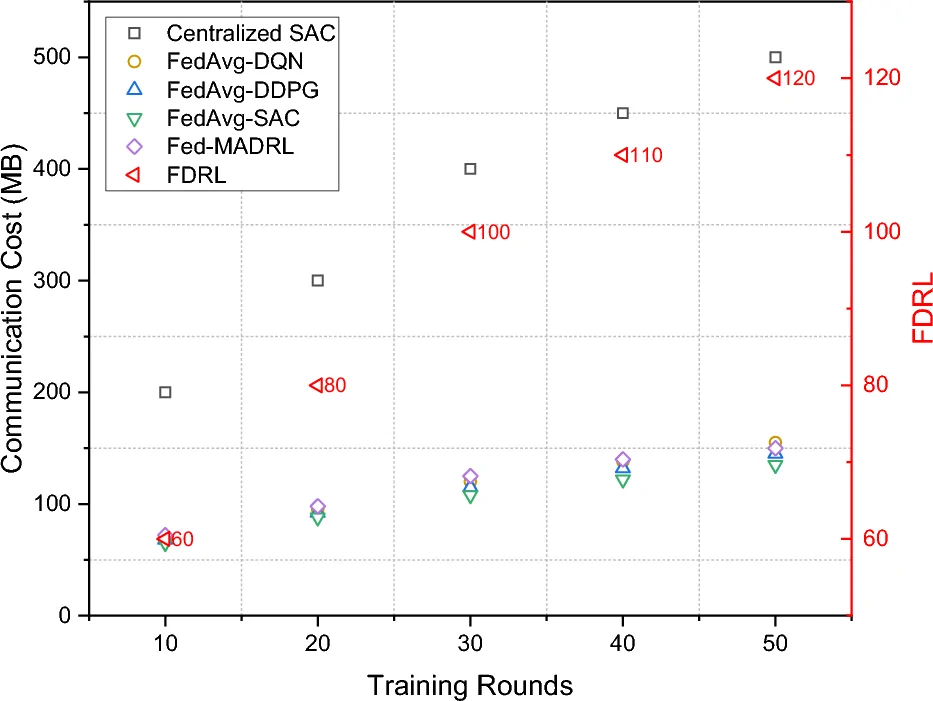
Communication overhead comparison between centralized SAC and federated baselines over 50 communication rounds.

We evaluate the effect of FL on communication overhead and learning performance, as summarized in Fig. 7 and Table 14. The analysis does not purport to provide formal privacy protection; rather, it investigates whether the proposed framework decreases centralized transmission of data by keeping raw data on vehicles at RSUs and exchanging only model parameters.

The centralized SAC baseline requires training on system-level observations, which increases communication costs and exposes raw vehicular data to the central learner. Contrarily, the federated baselines communicate model parameters at aggregation, leading to significantly reduced communication overhead. The proposed FDRL inherits the competitive learning performance while offering the added benefit of personalized aggregation, balancing global model sharing and local RSU adaptation under heterogeneous traffic, channels, and workloads.

Privacy-wise, the advantage of the proposed framework will only be limited to the raw-data locality. RSUs do not report raw vehicle paths, task logs, channel monitors, and mobility traces to the coordinator. However, the exchange of model parameters can still leak information by model inversion, gradient leakage, or membership inference attacks. Thus, the given framework must be understood as privacy-aware in minimizing the direct exposure of raw data, but should not be understood as a formally privacy-guaranteed procedure. Mechanisms for privacy that are officially considered privacy-enhancing, such as differential privacy, secure aggregation, and adversarial privacy testing, are also left as future work.

**Table 14:** Communication overhead and learning-performance comparison between centralized SAC and federated baselines over 50 communication rounds. For federated methods, the communication cost includes both the uplink model upload and the downlink global-model broadcast, using uncompressed 32-bit parameters.

Metric	C-SAC	FedAvg-DDPG	FedAvg-SAC	Fed-MADRL	FDRL
Communication cost over 50 rounds (MB)	500	145	135	150	120
Final reward	− 70	− 88	− 66	− 68	− 60
Convergence episodes	2500	3300	3000	3100	2800
Raw data transmission	Yes	No	No	No	No
Uplink + downlink included	Not applicable	Yes	Yes	Yes	Yes
Compression/quantization	No	No	No	No	No
Personalized aggregation	No	No	No	Partial	Yes

**Fig. 8:**
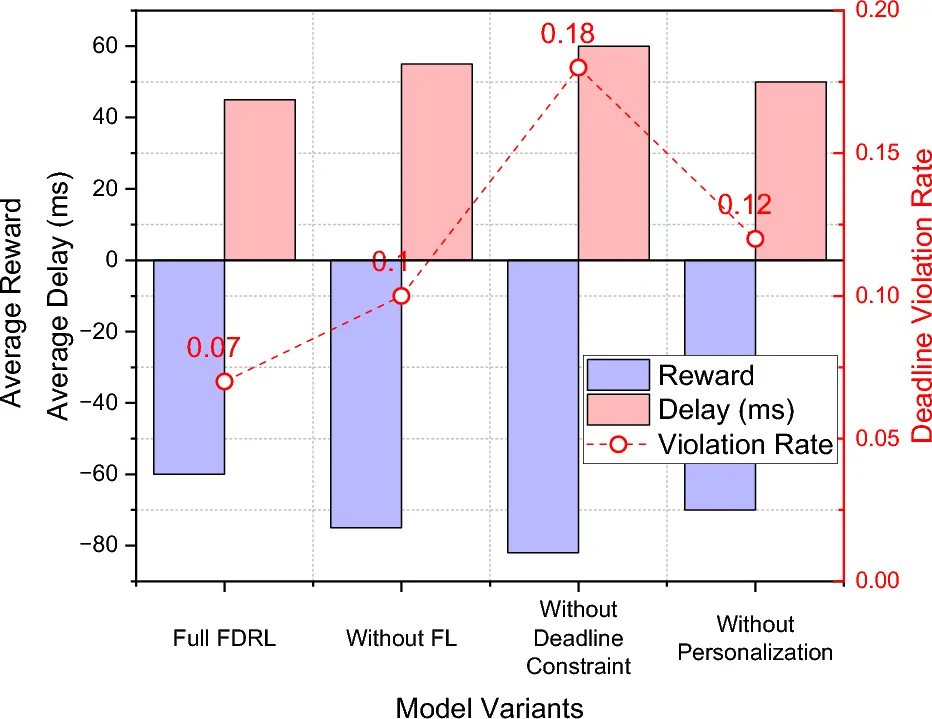
Ablation results showing the impact of FL, deadline-aware reliability modeling, and personalization on system performance. Results are reported as the mean over 30 independent runs.

### Ablation study

We conduct an ablation study to gauge the contribution of the key elements of the proposed framework as summarized by Fig. 8 and Table 15. The variants considered include the full FDRL model, FDRL without FL, FDRL without the deadline-aware constraint/reward component, and FDRL without the personalization mechanism.

Eliminating FL simplifies the system to locally trained RSU agents that cannot share collaboratively trained models. This undermines knowledge transfer across RSUs and diminishes resilience to heterogeneous traffic and channel conditions. Eliminating the deadline-aware element results in the greatest increase in the deadline-violation rate, indicating that deadline minimization, on its own, is insufficient in safety-sensitive vehicular applications. In the absence of the deadline penalty and the reliability constraint, the agent can decrease average cost while still allowing critical tasks to miss their deadlines.

The performance in the presence of non-IID RSU-level observations is also degraded by the removal of personalization. The tendency toward standard global aggregation drives all RSUs to a common policy, which might not suit local differences in mobility patterns, task arrival, channel quality, and resource availability. This trade-off is further enhanced by the personalization mechanism, which considers both global federated knowledge and local RSU adaptation. In general, the results of the ablation process indicate that the offered framework is performance-gaining due to the combined effects of FL, deadline-aware reliability modeling, and personalized aggregation, as opposed to its single-component nature.

**Table 15:** Ablation results showing the impact of federated learning, deadline-aware reliability modeling, and personalization on system performance.

Model variant	Reward	Delay (ms)	Violation rate
Full FDRL	− 60	45	0.07
FDRL without federated learning	− 75	55	0.10
FDRL without deadline-aware component	− 82	60	0.18
FDRL without personalization	− 70	50	0.12

### Sensitivity to personalization and non-IID heterogeneity

To measure personalization with heterogeneous Data in the form of RSU, we examine the impact of the personalization coefficient *β* and the non-IID distribution degree level. The coefficient *β* balances between the global federated model and the locally adapted RSU model: higher values give more weight in the global federated model, and lower values give more weight in the locally adapted RSU model.

We initially assess the proposed FDRL model under $\beta \in \{0,0.25,0.50,0.75,1.00\}$, which are provided in Table 16. Under *β =0*, the model becomes completely local and cannot leverage cross-RSU knowledge sharing. At *β =1*, the model becomes fully global and behaves similarly to regular federated averaging, potentially leading to decreased response to local non-IID conditions. Mid-range values will be more balanced between generalization in the world and specialization in the local area. The results indicate that *β =0.50* is the most suitable in the adopted simulation setting; it reduces system cost, delay, and the probability of violating the deadline compared to local-only and global-only options.

We then compare C-SAC, FedAvg-SAC, and the proposed FDRL in the scenario of mild, moderate, and heavy non-IID environments as summarized in the Table 17. With greater heterogeneity, the methods all degrade as RSUs experience a wider range of task distributions, traffic patterns, and channel conditions. However, C-SAC and FedAvg-SAC are more vulnerable to degradation because the former involves centralized training, while the latter uses only a global model and does not personalize it. Conversely, the suggested FDRL performs well in moderate and high non-IID environments, thanks to its use of both global and local knowledge/adaptation within the RSU. These findings justify the use of personalized federated aggregation in statistically heterogeneous vehicular edge environments.

**Table 16:** Sensitivity analysis of the personalization coefficient *β* under moderate non-IID conditions.

*β*	Interpretation	System cost	Avg delay (ms)	Violation rate
0.00	Local-only adaptation	152	52	0.12
0.25	Strong local personalization	142	48	0.09
0.50	Balanced global-local adaptation	135	45	0.07
0.75	Strong global influence	141	47	0.085
1.00	Global-only aggregation	145	49	0.10

**Table 17:** Effect of RSU-level statistical heterogeneity on centralized SAC, FedAvg-SAC, and proposed FDRL.

Heterogeneity level	Method	System cost	Avg delay (ms)	Violation rate
Mild non-IID	C-SAC	125	50	0.08
Mild non-IID	FedAvg-SAC	118	48	0.075
Mild non-IID	FDRL	112	45	0.065
Moderate non-IID	C-SAC	160	58	0.11
Moderate non-IID	FedAvg-SAC	145	52	0.09
Moderate non-IID	FDRL	135	45	0.07
High non-IID	C-SAC	195	70	0.16
High non-IID	FedAvg-SAC	178	62	0.13
High non-IID	FDRL	158	54	0.10

The results of these simulations indicate that the proposed FDRL framework is superior to the implemented centralized and federated baselines in the context of the adopted simulation setting. FL minimizes the transmission of raw data to a central location and ensures competitive learning outcomes; it should be understood, though, not as an explicit privacy guarantee but as an indication of decreased exposure to raw data. The ablation and sensitivity analyses further support the conclusion that the increase in performance is due to the synergistic effects of SAC-based hybrid action learning, deadline-sensitive reliability modeling, mobility-sensitive offloading, federated aggregation, and local personalization. The results aid the usefulness of the proposed design to control moderate-scale vehicular edge computing situations with dynamic workload and heterogeneous observations at the RSU level.

## Conclusion and future work

This study has introduced a privacy-aware FDRL model to offload a task in vehicular edge computing. The proposed architecture incorporates FL with local policy learning based on the SAC algorithm, allowing RSUs to train the offloading policy using locally observed data and to share model parameters but not the raw vehicular data. The framework addresses safety-sensitive IoV applications using task criticality, a deadline-reliable resource model, hybrid offloading and resource allocation, fine-grained handover delay, and customized federated aggregation for RSU-level environments that are non-IID.

Simulation results show that the proposed framework has lower system cost, task delay, energy consumption, deadline reliability, and communication overhead than the installed centralized and federated baselines in the adopted simulation environment. The findings also indicate that FL minimizes the transmission of raw data to a centralized location; however, this privacy consideration can be viewed as a reduction in the minimization of raw data to a centralized point, rather than an explicit guarantee of privacy. The performance gain can also be confirmed with the help of the ablation and sensitivity analysis, which indicated that the performance gain was the combined effect of SAC-based hybrid action learning, deadline-aware reliability modeling, mobility-aware offloading, federated aggregation, and local personalization.

Future work will extend the evaluation to larger city-scale scenarios using SUMO-generated and real-world urban mobility traces with more realistic RSU deployment. In the communication model, the interference-aware V2I link, the effects of packet-level errors and retransmissions, and uncertainty in channel estimation will also be incorporated to improve it. Furthermore, the future study will investigate privacy and robustness issues using model inversion and membership inference attacks, establish convergence results for personalized federated off-policy reinforcement learning under non-IID vehicular dynamics, and investigate model compression and communication-efficient aggregation when a large number of RSUs are installed. Where possible, simulation scripts, random seed settings, and configuration files to aid reproducibility will be shared.

## Data Availability

The datasets used and analyzed during the current study are available from the corresponding author on reasonable request.
